# Knee joint biomechanics and cartilage damage prediction during landing: A hybrid MD-FE-musculoskeletal modeling

**DOI:** 10.1371/journal.pone.0287479

**Published:** 2023-08-03

**Authors:** Malek Adouni, Fadi Alkhatib, Afif Gouissem, Tanvir R. Faisal

**Affiliations:** 1 Physical Medicine and Rehabilitation Department, Northwestern University, Chicago, IL, United States of America; 2 Mechanical Engineering Department, Australian University, East Mushrif, Kuwait; 3 Department of Mechanical Engineering, University of Louisiana at Lafayette, Lafayette, LA, United States of America; University of Memphis, UNITED STATES

## Abstract

Understanding the mechanics behind knee joint injuries and providing appropriate treatment is crucial for improving physical function, quality of life, and employability. In this study, we used a hybrid molecular dynamics-finite element-musculoskeletal model to determine the level of loads the knee can withstand when landing from different heights (20, 40, 60 cm), including the height at which cartilage damage occurs. The model was driven by kinematics–kinetics data of asymptomatic subjects at the peak loading instance of drop landing. Our analysis revealed that as landing height increased, the forces on the knee joint also increased, particularly in the vastus muscles and medial gastrocnemius. The patellar tendon experienced more stress than other ligaments, and the medial plateau supported most of the tibial cartilage contact forces and stresses. The load was mostly transmitted through cartilage-cartilage interaction and increased with landing height. The critical height of 126 cm, at which cartilage damage was initiated, was determined by extrapolating the collected data using an iterative approach. Damage initiation and propagation were mainly located in the superficial layers of the tibiofemoral and patellofemoral cartilage. Finally, this study provides valuable insights into the mechanisms of landing-associated cartilage damage and could help limit joint injuries and improve training programs.

## Introduction

High degrees of physical activity, such as drop landing, may lead to acute joint injury, especially if it is associated with elevated height. The joint alteration, including significant ligaments and cartilage damage, ultimately leads to degenerative joint disease such as osteoarthritis (OA) and, consequently, joint failure [1]. Understanding landing joint loading to prevent soft tissue damage is important but not well-defined. We argue that the development of such a concept can best be accomplished by understanding the relationship between external loading and the basic molecular structure of soft tissue, starting at the fibril level. Identifying the nature of the connection between damage at the fibril level, defined here as micro-defects, and soft tissue loading can help elucidate the biochemical and mechanical interactions between aggregate landing loading levels and acute joint injury. However, exploring these interactions under experimental conditions in man or animals is technically prohibitive [2]. Thus, computational biomechanical modeling is considered a vital complementary tool to improve our knowledge of joint response.

Computational models associated with experimental measurements have previously been used to estimate muscular and joint forces for a number of functional tasks, including drop landing [3–14]. These models have numerous clinical applications as well as been widely used in rehabilitation medicine. However, the current musculoskeletal models only consider whole-body kinematics to describe the macro-mechanics without integrating the microscopic details and the capacity of soft tissues [9]. This capacity has been well documented as mechanical failure states mainly depend on molecular characteristics [15]. At one extreme, the dynamic molecular syntheses start from the amino acid molecule and limit to the smallest macro unity of tissues (extracellular matrix) (e.g. [16]) and treat this unity as a load receptor, independent of the actual macro-mechanics. At the other extreme, current multi-body inverse dynamic simulations targeting muscles and joints loading, which use measured kinematics and ground reaction forces as input (e.g. [9]), treat joints as kinematic constraints that undergo the same motion irrespective of the external loading conditions. To date, the efficacy of such a study regarding the ligaments and cartilage damage initiation and propagation was limited to in vitro testing or macro sub-modeling tools [17]. Here, a paradigm shift in kinematics measurement and musculoskeletal simulation by accounting for the interplay between skeletal dynamics and micro internal joint mechanics, which is fundamental to addressing soft tissue overusing injuries, degenerative joint disease, and osteoarthritis (OA), is missing.

An accurate understanding of soft tissue failure initiation could be comprehended by simultaneously combining actual and extrapolated kinematics/kinetics measurements with a multiscale computation paradigm that links the molecular foundation of the soft tissue to the continuum level [3, 18]. Therefore, a hybrid computational framework linking three different syntheses—molecular dynamics (MD), finite element analysis (FEA), and musculoskeletal modeling was developed. This computational construct was used to evaluate critically the landing biomechanics of the knee joint as a function of height, one of the most associated factors with cartilage injury, as well as cartilage damage prediction. This evaluation may permit a better understanding of the mechanisms underlying soft tissue damage initiation and its spatial propagation.

## Methods

### i) Kinematics-driven model

**Fig 1:**
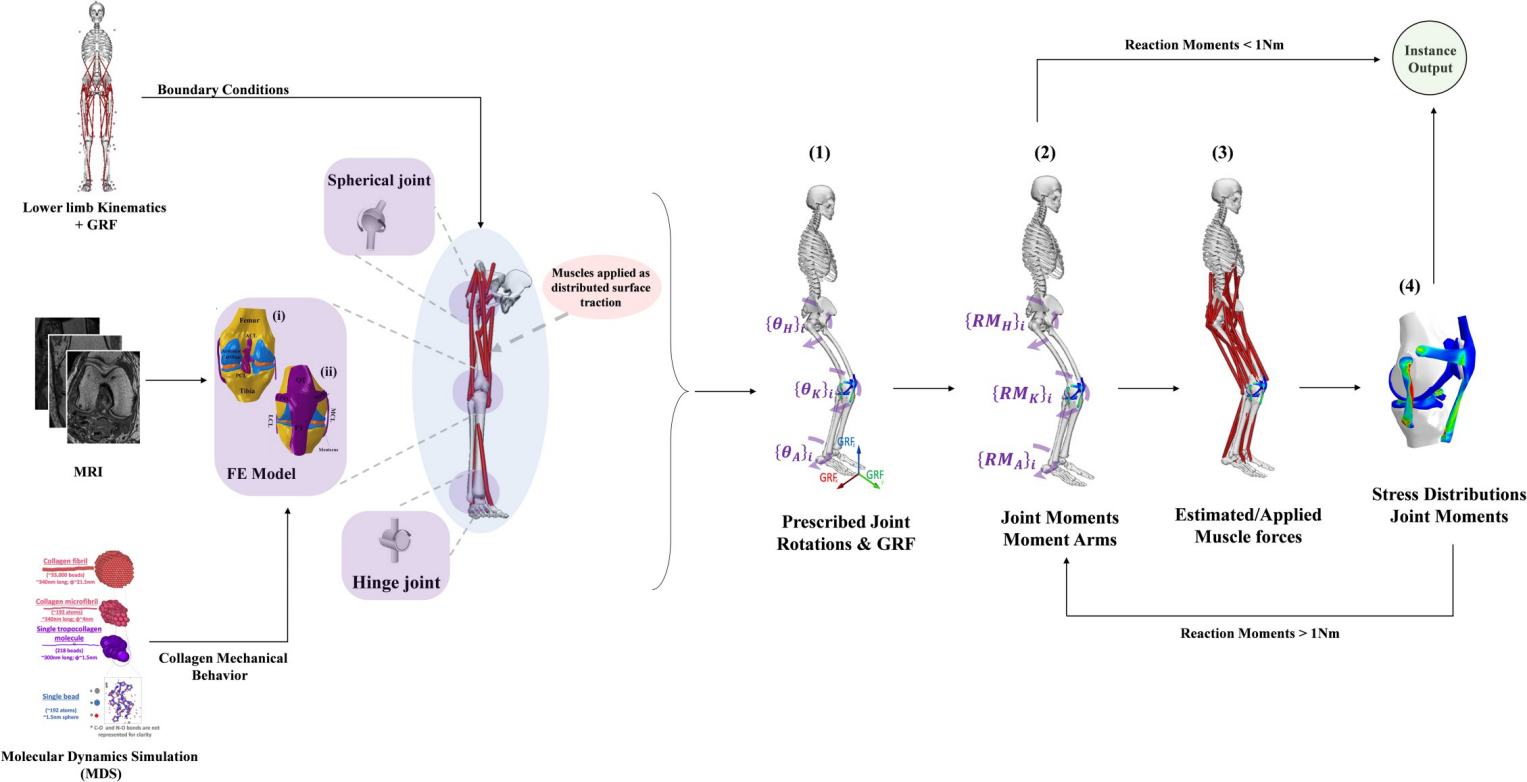
The adopted workflow includes a 3D finite element model of the knee viewed from the anterior (i) and posterior (ii) perspectives. (1) Prescribed joint rotations and GRF, (2) Computing joint reaction moments and muscle moment arms, (3) estimated muscle forces and applied as surface tractions [28], (4) Computing joint stress and updated reaction moments. For more information regarding the system of axes, joint center calculations, and muscle characteristics, please refer to [9, 28].

A kinematics-driven musculoskeletal model of the lower extremity accounts for the hip and ankle as spherical and hinge joints, respectively, and the knee as FE model, as well as their active musculatures (34 muscles), was developed. The knee model was reconstructed from a digitized MRI (OpenKnee public domain repository at Simtk.org) scanned at Cleveland Clinic (Biomechanics laboratory) for 70 years female subject (Weight = 77 kg and Height = 170 cm) using a Tesla extremity MRI scanner (Orthone, ONI Medical Systems-Inc, Wilmington-MA) [11]. These image data were manually segmented and re-sampled in the anatomical planes using 3D Slicer 4.8 (viewing and segmentation analysis package). Geometrical surface smoothness, correction, and mesh generation were conducted via SolidWorks (SolidWorks Corp., Concord, MA, USA) and HyperMesh pre-processor (Altair Engineering, Troy, MI). The geometry of the tibiofemoral joint was adjusted to align with the dimension reported in the Simtk.org open knee public domain repository [11]. In the model, bones were represented as rigid bodies [19–23] utilizing shell elements (S4R), whereas articular cartilages, ligaments, and menisci were depicted through reduced integration brick elements (C3D8R), as shown in Fig 1. Details of the knee model were presented in the S1 File and our prior works [24–27].

### ii) Constitutive models of the soft tissue

#### a) Collagen mesoscopic model

The purpose of the MD simulation is to obtain the micromechanical behavior of collagen fibrils. The simulations are based on a mesoscopic model proposed by Buehler [29]. The concept of the mesoscopic model is to abbreviate the full molecular geometry of the collagen molecule (consisting of three chains of amino acids and having a diameter of about 1.6 nm and a length of about 300 nm) into a single chain of beads (or super atoms) where each bead represents several atoms in the full atomistic model [16, 30–32]. This approach allows molecular dynamics to reach time and length scales otherwise inaccessible by simulating full molecular structures. The fibril is then built by replicating the above-described molecule orthogonally to its principal axis [33] in a quasi-hexagonal array where each group of 5 molecules packs together to form a microfibril. In the present work, we assumed a fibril diameter of 21.5 nm containing 151 molecules. The formulation of the coarse-grained model of collagen molecules was implemented in several studies [31, 34, 35] and was proven to accurately mimic the actual behavior of the fibril. The force field is governed by three main energies as follows:

$$\left\{\begin{array}{c} E_{inter}\;=\;4\varepsilon \left({\left(\frac{\sigma }{r}\right)}^{12}-\;{\left(\frac{\sigma }{r}\right)}^{6}\right)\;\\ E_{bond}\;=\;\left\{\begin{array}{cc} \frac{K_{T0}}{2}{\left(r-r_{0}\right)}^{2}\;+\;C_{1} & ,r\;<\;r_{1}\\ \frac{K_{T1}}{2}{\left(r-\bar{r_{1}}\right)}^{2}\;+\;C_{2} & ,r_{1}\;<\;r\;<\;r_{b}\\ 0 & ,r\;>\;r_{b} \end{array}\right.\\ E_{angle}\;=\;K_{\theta }{\left(\theta \;-\;\theta_{0}\right)}^{2}\; \end{array}\right.\;$$ (1)

Where *E*_*inter*_, *σ*, and *ε* represent, respectively, the interatomic energy, characteristic distance, and the minimum energy of the Lennard Jones potential. *E*_*bond*_, *K*_*T*0_, and *K*_*T*1_, are the bond energy and spring constants, respectively, *r*_1_, $\bar{r_{1}}$, *r*_*b*_ are the distances of the hyper-elastic behavior, continuity of the force field, and breaking bond, respectively. *E*_*angle*_ and *K*_θ_ are the angular energy and the bending strength, respectively, *θ*_0_ and *θ* are the equilibrium and actual angle between the three consecutive beads. Enzymatic crosslinks are then added to the fibril model by bonding telopeptides and helical residues from adjacent molecules. The coefficient β represents the density of molecule ends that are connected to beads from other molecules (a coefficient β = 100% corresponds to 2 connected ends per molecule). The fibril model was created using MatlabR2021A by averaging the geometric positions of the atoms in the 3HR2 PDB (Collagen I) entry and replicating the molecule in the radial directions. All MD simulations were performed using LAMMPS molecular dynamics software [36] (Fig 1). Additional details about the developed model can be found in the S1 File and our prior works [18, 24, 37, 38].

#### b) Cartilage

To simulate collagen fibril behavior, a nonlinear constitutive modeling approach developed by Sajjadinia et al., [39] in which the stress of the fibril can be defined as:

$$\left\{\begin{array}{c} \sigma_{i}^{f}\;=\;{\left\{\frac{\eta_{0}^{s}}{J}\;ln\varepsilon_{f}\left(E_{0}\varepsilon_{f}\;+\;E_{\varepsilon }\varepsilon_{f}^{2}\right)(n⊗n)\right\}}_{i}\;\varepsilon_{f_{i}}\;≻\;0\\ \sigma_{i}^{f}\;=\;0\;\varepsilon_{f_{i}}\;≼\;0 \end{array}\right.$$ (2)

Where *n* and *ε*_*f*_ are the current direction and logarithmic strain of the fibril, respectively. *E*_0_ and *E*_*ε*_ are the collagen stiffening coefficients (initial and strain-dependent) and $\eta_{0}^{s}$ is a depth-dependent elastic material parameter. The collagen networks were categorized into primary and secondary bundles (*i*) of fibrils, based on their orientation in relation to the depth of the articular cartilage. The primary fibrils were aligned perpendicular to the subchondral junction and gradually rotated in the middle zone to become parallel to the articular surface. The integration of the fibril stress equation with respect to strain in its axial form led to the strain-energy function (*W*_*fl*_) [40]. The softening hyperelasticity approach for modeling nonlinear materials failure [40–43] has been considered in this work to unify the nonlinear elasticity with plastic (failure) descriptions. The softening of the fibrils was captured by a constant *Ф* (*energy limiter*) [44–46]. Thereafter, the strain energy function of the fibril is modified with the inclusion of the energy limiter and takes the following form:

$$\psi \left(\Phi ,W_{fl}\right)\;=\;\frac{\Phi }{m}\;\left\{\Gamma \left(\frac{1}{m},0\right)\;-\;\Gamma \left(\frac{1}{m},\;\frac{W_{fl}^{m}}{\Phi^{m}}\right)\right\}$$ (3)

where *Γ* is the upper incomplete gamma function expressed as $\Gamma (s,x)\;=\;\int_{x}^{\infty }t^{s-1}exp\;(\;-\;t)dt,\;W_{fl}$ is the strain energy of the intact (without failure) fibril, and the dimensionless material parameter *m* controls the sharpness of the transition of material softening. Differentiating the modified strain energy [47] yields the following fibril stress under uniaxial tension,

$$\sigma_{i}^{fl}\;=\;\sigma_{i}^{f}exp\left(\;-\;\frac{W_{fl}^{m}}{\Phi^{m}}\right)$$ (4)

The cartilage was modeled with incompressible hyperelastic behavior, strengthened by the newly developed continuum-damage model of the fibril. The Cauchy stress (*σ^c^*) in the used model was decomposed into a non-fibrillar (*σ*^*nf*^) and fibrillar ($\sigma_{i}^{fl}$) parts as follow:

$$\left\{\begin{array}{c} \sigma^{c}=v_{f}\sigma^{fl}+\left(1-v_{t}\right)\sigma^{nf}\;\;\;\\ \sigma^{nf}=\eta_{0}^{s}\left[-\frac{\ln J}{6J}G_{m}\left(\frac{3\eta_{0}^{s}lnJ}{\eta_{0}^{s}-1}-3\frac{J+\eta_{0}^{s}}{J-\eta_{0}^{s}}-1\right)I+\frac{G_{m}}{J}\left(FF^{T}-J^{2/3}I\right)\right]+\frac{1}{D}{\left(J-1\right)}^{2}\;\\ \sigma_{i}^{fl}=\sigma_{i}^{f}\exp\left(-\frac{W_{fl}^{m}}{\Phi^{m}}\right)\;\;\varepsilon_{f_{i}}≻0\;\\ \sigma_{i}^{fl}=0\;\;\varepsilon_{f_{i}}≼0\; \end{array}\right.$$ (5)

Here, *F* represents the deformation gradient tensor, and *J* represents the volumetric deformation. G_m_ is the shear modulus and *v*_*f*_ is the relative collagen fibril volume fraction. Based on a 30% reduction of stiffness of collagen II compared with collagen I and the similarity of a nonlinear trend reported in certain experimental and theoretical investigations [48–50], the parameters driving the fibril response of the articular cartilage were calculated by fitting them to 70% of the predicted MD simulation results (Collagen Mesoscopic Model). The rest of the parameters were fixed based on the earlier investigation of Sajjadinia et al., [39]. For further information on the material formulation, please refer to the S1 File and previous publications [39, 51]. A list of the properties of the material is presented in Table 1.

**Table 1:** Articular cartilage materials properties obtained from MD fitting and earlier investigation of Sajjadinia et al., [39].

Material parameters	
***E*_*0*_(*MPa*)**: Initial collagen coefficients	8.121
***Eε*(*MPa*)**: Strain-dep collagen coefficients	5326.32
**ϕ**: Energy limiter	82.326
***m***: dimensionless material parameter	12
***G*_*m*_ (*MPa*)**: Shear modulus	0.723
***v*_*f*_**: Collagen fibril volume fraction^1^	*$v_{t}\frac{3}{13}$ or $v_{t}\frac{1}{13}$*
***v*_*t*_**: Total depth-dependent collagen volume fraction^2^	1.4*Z*^2^ ‒ 1.1z + 0.59
$\eta_{0}^{s}$: Elastic material parameter	0.1*z* + 0.1
***D***: Incompressibility penalty parameter	0.0001

^1^$\frac{3}{13}\;\;\text{and}\;\;\frac{1}{13}$ for the primary and the secondary fibril, respectively.

^2^z: The depth of the articular cartilage, measured from the junction of the cartilage and bone and normalized accordingly.

#### c) Meniscus

A specific class of materials, transverse isotropy, was used to represent the aggregate behavior of the meniscus [23, 49, 52–54]. Three axes (axial, transversal, circumferential) were defined to identify the local material orientation considering the isotropy of the transverse-axial plane. As a result, the stiffness matrix linking meniscus stress-strain was described as follows:

$$\left\{\begin{array}{l} \varepsilon_{11}\\ \varepsilon_{22}\\ \varepsilon_{33}\\ \gamma_{12}\\ \gamma_{13}\\ \gamma_{23} \end{array}\right\}\;=\;\left[\begin{array}{cccccc} 1/Et & -\vartheta \text{ct}/\text{Et} & -\vartheta \text{ta}/\text{Ec} & 0 & 0 & 0\\ -\vartheta \text{ct}/\text{Et} & 1/Et & -\vartheta \text{ta}/\text{Ec} & 0 & 0 & 0\\ -\vartheta \text{ta}/\text{Et} & -\vartheta \text{ta}/\text{Et} & 1/Ec & 0 & 0 & 0\\ 0 & 0 & 0 & 1/G & 0 & 0\\ 0 & 0 & 0 & 0 & 1/G & 0\\ 0 & 0 & 0 & 0 & 0 & 1/G \end{array}\right]\;\left\{\begin{array}{l} \sigma_{11}\\ \sigma_{22}\\ \sigma_{33}\\ \sigma_{12}\\ \sigma_{13}\\ \sigma_{23} \end{array}\right\}$$ (6)

Where *E*_*C*_ is the circumferential modulus, *E*_*t*_ and *E*_*a*_ are transverse and axial modulus (*E*_*t*_ *= E*_*a*_), respectively, *ν*_*ct*_ and *ν*_*ca*_ are the Poisson’s ratio, which is defined as the ratio of the contractile strain in the transverse plane to the tensile strain in the circumferential direction under the load in the circumferential direction (*ν*_*ct*_ *= ν*_*ca*_), *ν*_*ta*_ is the Poisson’s ratio within the transverse plane and G is the shear modulus. A list of the properties of the material is presented in Table 2.

**Table 2:** Meniscus materials properties [5,5].

E_c_ (MPa)	E_t_ (MPa)	*v*_*ct*_	*v*_*ta*_	G_t_ (MPa)
120	20	0.3	0.2	47

#### d) Ligaments

*Ligaments pre-strains*. The total deformation gradient was decomposed into the reference (*F*) and stress-free states (*F*_*0*_) in which the ligaments pre-strains were introduced via initial stretch (*α*_0_) as follows,

$$F_{0}\;=\;\left[\begin{array}{ccc} \alpha_{0} & 0 & 0\\ 0 & \sqrt[{-1}]{\alpha_{0}} & 0\\ 0 & 0 & \sqrt[{-1}]{\alpha_{0}} \end{array}\right]$$ (7)

For more details about the considered initial stretch, please see our recent publication [55].

*Ligaments model*. The patellar tendon and ligaments were considered as a homogenized continuum set of elements with a hierarchical concept of fibrils and fiber reinforcement. This concept assumes an incompressible hyper-elastoplastic behavior with a plastic flow associated with the uniaxial tension of the collagen fibrils. Therefore, the total deformation gradient tensor was decomposed into elastic and plastic parts ($\bar{F}\;=\;{\bar{F}}_{e}{\bar{F}}_{p}$) with the following invariants ${\bar{I}}_{le}\;=\;tr\left({\bar{C}}_{\text{e}}={\bar{F}}_{\text{e}}\right.\;\left.{\bar{F}}_{\text{e}}^{\text{T}}\right)\;\text{and}\;{\bar{I}}_{4e}\;=\;n_{o}{\bar{C}}_{e}n_{o}^{t},{\bar{I}}_{1ef}\;=\;{\bar{I}}_{4}\;+\;2{\bar{I}}_{4}^{-1/2}$ with C is the Cauchy-Green tensor and *n*_*o*_ is the initial orientation of the collagen fibril [56, 57]. Then, a mixed pyramidical formula was employed, in which the collagen fiber reinforced the ligaments, and the fibril reinforced the fiber itself. As a result, the total strain energy density combining an extension (e) and shear (s) behavior is given by:

$$\begin{array}{c} \psi_{t}\left({\bar{I}}_{1f},{\bar{I}}_{4},{\bar{I}}_{4e}\right)={\left[v_{f}\psi_{fi}\left({\bar{I}}_{1f},{\bar{I}}_{4},{\bar{I}}_{4e}\right)+v_{m}\left(\frac{\mu_{m}}{2}\left({\bar{I}}_{1}-3\right)\right)\right]}_{e}+{\left[\frac{1}{2}\mu_{m}\frac{\left(1+v_{f}\right)\mu^{efffb}\left({\bar{I}}_{4e}\right)+\mu_{m}\left(1-v_{f}\right)}{\left(1-v_{f}\right)\mu^{efffb}\left({\bar{I}}_{4e}\right)+\mu_{m}\left(1+v_{f}\right)}\left({\bar{I}}_{1}-{\bar{I}}_{1f}\right)\right]}_{s}+ \end{array}\psi_{vol}(\bar{J})$$ (8)

Where the fiber strain energy is given by:

$$\psi_{fi}\left({\bar{I}}_{1f},{\bar{I}}_{4},{\bar{I}}_{4e}\right)={\left[v_{fb}\psi_{fb}\left({\bar{I}}_{1ef},{\bar{I}}_{4e}\right)+v_{mb}\left(\frac{\mu_{fm}}{2}\left({\bar{I}}_{1ef}-3\right)\right)\right]}_{t}+\left[\frac{1}{2}\mu_{fm}\frac{\left(1+v_{fb})\mu^{fb}({\bar{I}}_{4e})+\mu_{0}(1-v_{fb}\right)}{\left(1-v_{fb})\mu^{fb}({\bar{I}}_{4e})+\mu_{0}(1+v_{fb}\right)}\left({\bar{I}}_{1e}-\right.{\left.\left.{\bar{I}}_{1ef}\right)\right]}_{s}\right.$$ (9)

And the fibril strain energy is given by:

$$\psi_{fb}\left({\bar{I}}_{1e},{\bar{I}}_{4e}\right)=\frac{1}{2}\mu_{o}\left(tanh\left(a_{1}\left({\bar{I}}_{4e}-1\right)\right)+a_{2}\;exp\left(a_{3}\left({\bar{I}}_{4e}-I_{o}\right)\right)\right)\left({\bar{I}}_{1ef}-3\right)$$ (10)

Where *μ*_*o*_, *I*_*o*,_ *and a*_*i*,_ represent the shear modulus, secondary stiffening, and the dimensionless parameters of the fibril model, respectively.*μ*_*fm*_,*μ*_*m*_, *v*_*mb*_, *v*_*m*_, *v*_*fb*_, and *v*_*f*_ are the shear moduli of the fiber and tissue matrices, the volume fraction of the fiber and tissue matrices, and the volume fraction of the fiber and the fibrils, respectively. Under the constraints of soft tissue incompressibility and Clausius-Duhem dissipation inequality, the total stress (*σ*_*t*_) of soft tissue was characterized by fibrillar *σ*_*f*_ and nonfibrillar *σ*_*nf*_ stress tensors, as shown below.


$$\left\{\begin{array}{cccc} \sigma_{t}=\sigma_{nf}+\sigma_{f} & & & \\ \sigma_{nf}=\frac{2}{J}\left({\bar{I}}_{1}\frac{\partial \psi_{t}}{\partial {\bar{I}}_{1}}dev(\bar{B})+\left(E_{k}\bar{J}(\bar{J}-1)\right)I\right) & & & \\ \sigma_{f}=\frac{2}{J}\left({\bar{I}}_{4}\frac{\partial \psi_{t}}{\partial {\bar{I}}_{4}}dev(n⊗n)+{\bar{I}}_{4e}\frac{\partial \psi_{t}}{\partial {\bar{I}}_{4e}}dev\left(n_{e}⊗n_{e}\right)\right) & & & if\;{\bar{I}}_{4}|>1\\ \sigma_{f}=0 & & & if\;{\bar{I}}_{4}|\leq 1 \end{array}\right.$$ (11)

The stress-strain function of the fibrils drove the elasto-plastic behavior using the single crystal plasticity model under the Karush-Kuhn-Tucker loading/unloading constraint [58–60]. This stress-strain function was obtained from our MD simulations results (Collagen Mesoscopic Model). Fibril parameters were fixed based on the output of the MD simulation, and the rest of the ligaments and tendon parameters were calibrated using a statistical approach to fit the aggregate mechanical response of the soft tissues [24]. Additional details of the developed model can be found in the S1 File and our prior works [18, 24, 61].

### iii) Muscles optimization

The lower extremity muscle forces ({*x*}) were optimized via a nonlinear static optimization technique at the peak loading instance of the drop landing phase. Along with physiological muscle limitations (muscle forces between the passive and total maximum active forces (Eq 14)), the main driver of this optimization was the joint equation of equilibrium (Eq 13), where the sum of cubed muscle stresses was minimized as an objective function (Eq 12). The muscle boundaries were determined by using a scaled musculoskeletal model that corresponded to the subject’s dimensions, which was utilized to construct the kinematics-driven model [9].


$$f\left(x_{i}\right)=\sum_{i}^{n}{\left(\frac{x_{i}}{PCSA_{i}}\right)}^{3}$$ (12)

[R]{x}={M} (13)

$$\left\{x_{p}\right\}\leq \{x\}\leq \left\{x_{max}\right\}$$ (14)

[*M*] are the required lower extremity joint moments computed at the peak loading instance of the drop landing phase and *x*_*p*_, *x*_*mas*_, [R], *PCSA*_*i*_, *x*_*i*_ are the passive and maximum components of muscle force, lever arms matrix, physiological cross-sectional areas and force of muscle *i*, respectively [9].

### iv) Drop landing data and regression equations

A detailed search was performed to determine published journal articles reporting drop landing with both legs (peer-reviewed in English) with a publication range between 1990 and 2021. Irrelevant publications were initially excluded based on rapid screening of the title and abstracts and second on a detailed inspection of the full text when the first screening provided insufficient information. Finally, only 42 articles have been selected (_((((xxx))))_)[62–104]. The following criteria were considered during the selection process: (1) Investigation was performed on healthy adult humans (14–38 yr.) (Table 3). (2) Subjects regularly drop with both legs. (3) Drop height was reported. (4) Lower limb kinematics/kinetics at the peak of ground reaction forces were reported.

**Table 3:** Relevant subjects’ information [62–104], data are presented as mean (standard deviation).

Age (years old)	22.9 (3.1)
Weight (kg)	66.2 (7.2)
Height (cm)	171.3 (5.4)
BMI (kg/m^2^)	22.4 (1.2)

**Fig 2:**
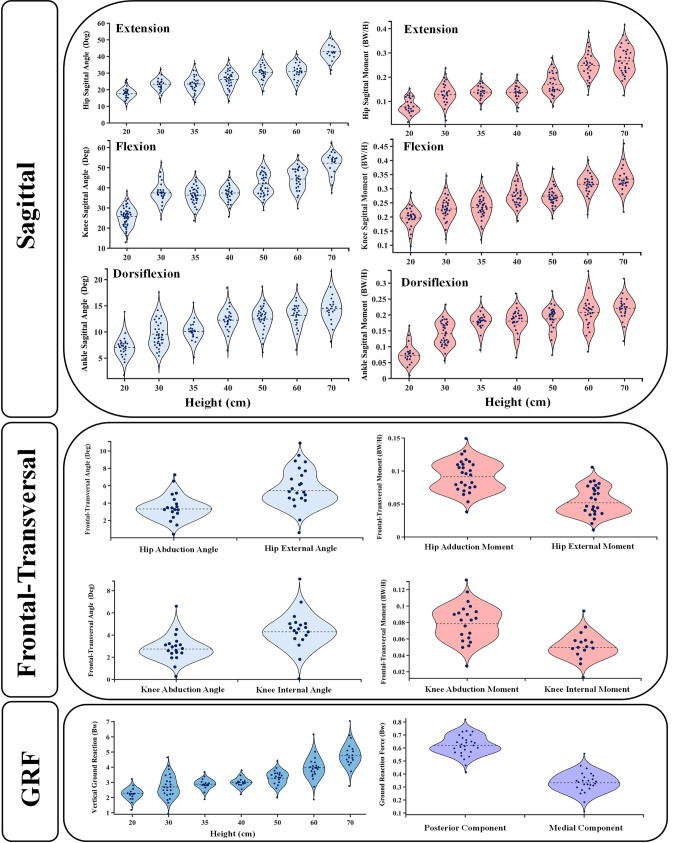
Violin plot showing lower limb joints kinematics and kinetics data distributions with the dashed line representing the mean value. (a) sagittal joints angles distributions for different drop landing heights, (b) sagittal joints moments distributions for different drop landing heights, (c) frontal-transversal joints angles distributions, (d) frontal-transversal joints moments distributions, (e) vertical GRF distributions for different drop landing heights (f) Posterior-Medial GRF distributions, all collected at the peak loading instance based on experimental data.

To determine the kinematics/kinetics and ground reaction forces of supra-physiological impact, also known as a large landing height analysis that is inaccessible via drop landing analysis, a regression relationship between landing height and these parameters were developed. Here, the sagittal lower limb kinematics/kinetics and vertical ground reaction forces were considered dependent variables to the landing height (Fig 2). Nonlinear regression equations involving power, exponential, and natural logarithmic were used to fit experimental data. A strong regression relationship was identified by an *R*^*2*^ value near one (Table 4). The coupled kinematics/kinetics (frontal-transversal planes) was excluded from the regression process due to the low dependency of these parameters on the landing height [62, 80] (Fig 2). Hence, the means of these parameters were used with different heights during the simulations. Finally, as verification, similar output to experimental measurements [105] of a height of around 1 m of these regression models was computed.

**Table 4:** Non-linear regression equations and corresponding regression coefficients obtained from experimental data measured at the peak loading instance of drop landing analysis [6,2–10,4].

Dependent variables	Regression equations	Regression coefficients		*R* ^*2*^
		*a*	*b*	
**Hip flexion angle**	*y* = *ax*^*b*^	3.3896	0.557	0.9418
**Knee flexion angle**	*y* = *aln*(*x*) + *b*	14.769	-15.237	0.9082
**Ankle dorsiflexion angle**	*y* = *ae*^*bx*^	8.1108	0.0068	0.9811
**Hip flexion moment**	*y* = *ax*^*b*^	0.0096	0.7563	0.9486
**Knee flexion moment**	*y* = *ax*^*b*^	0.0699	0.3704	0.9129
**Ankle dorsiflexion moment**	*y* = *aln*(*x*) + *b*	0.1086	-0.2315	0.9187
**Vertical GRF**	*y* = *ax*^*b*^	0.4468	0.542	0.9463

### v) Loading and boundary conditions

The nonlinear regression models (sagittal) and the average of coupled data (frontal-transversal) of hip-knee-ankle joints kinematics and kinetics (Fig 2 and Table 4) at the peak ground reaction instance were used to drive the musculoskeletal model. First, the analyses were performed at three different landing heights (20 cm, 40 cm, and 60 cm). Then, one additional analysis treating cartilage damage initiation was performed based on a trial-error test (height = 126 cm). The femur was immobilized in its instantaneous position, while the tibia was subjected to prescribed joint rotations, and the patella was left unconstrained. To recreate the joint reaction moments, the weight of the leg/foot and the ground reaction force were applied, and the unknown muscle forces were calculated iteratively. The iteration was conducted based on applying muscle forces as additional external loads to the model; this updated the optimization algorithm with residual reaction moments and muscle lever arms, which resulted from passive resistance [3, 55, 106–108]. The convergence was achieved when the required moments fell below 1 N.m (Fig 1). Matlab genetic algorithm and Abaqus quasi-static analysis were used for the optimization. Please see our earlier publication [55] for more details about the muscles optimization algorithm.

## Results

**Fig 3:**
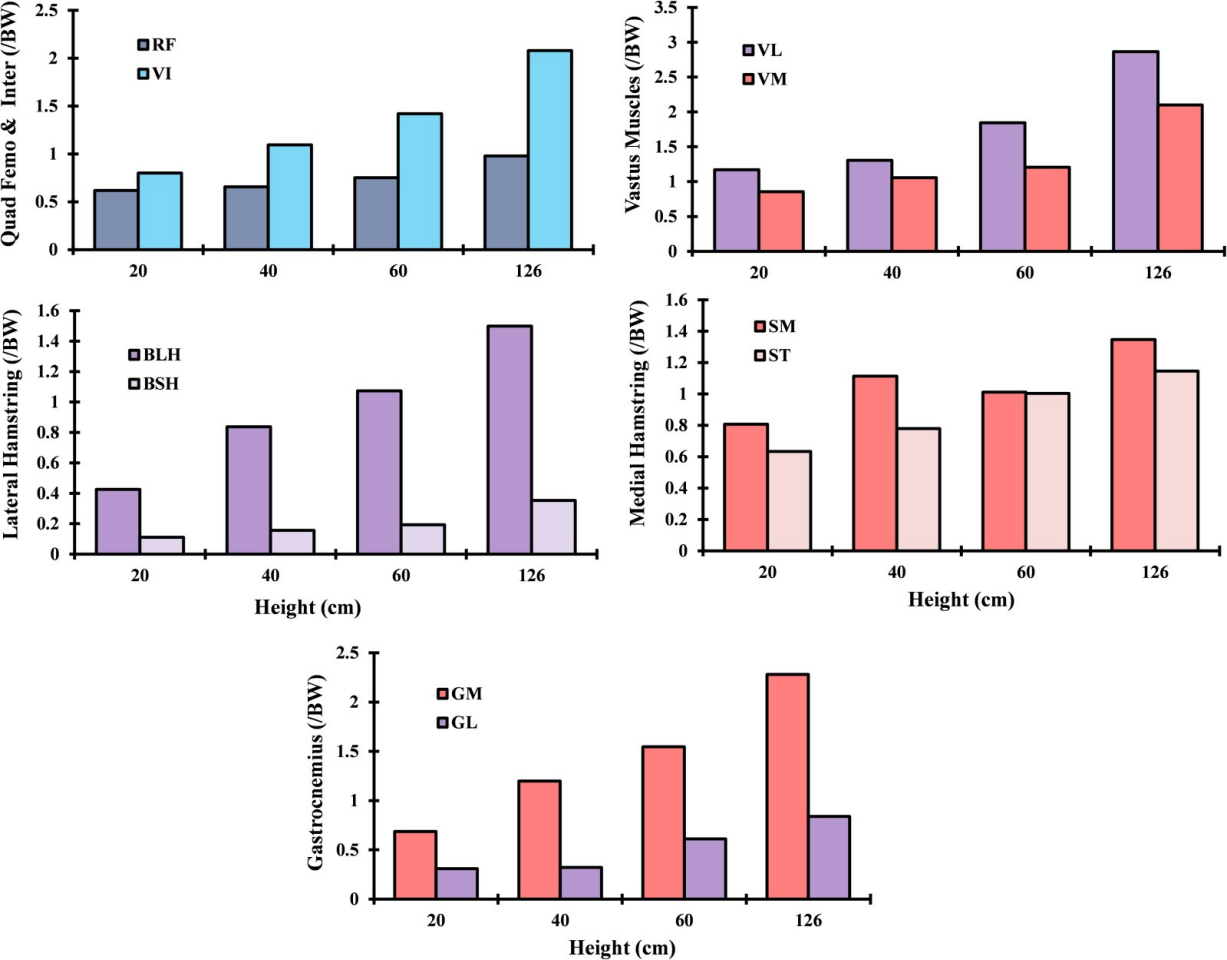
Computed muscle forces for different drop landing heights at the peak loading instance. Quadriceps: Vastus intermedius (VI), Vastus medialis (VM), Rectus femoris (RF), and Vastus lateralis (VL); Gastrocnemius: medial (MG) and lateral (LG); Hamstrings: Semimembranosus (SM) and Semitendinosus (ST), Biceps femoris long and short head (BLH, BSH).

As shown in Fig 3, the muscle forces in the quadriceps demonstrated a significant increase, averaging 42%, when the height of the drop landing was raised from 20 to 60 cm and continued to increase by approximately 33% when the height was raised to the supraphysiological level of 126 cm. The vastus intermedius and vastus lateralis were significantly more affected, with their activity increasing by more than two-fold when exposed to a drop landing height of 126 cm. Furthermore, the medial hamstring (Semimembranosus (SM) and Semitendinosus (ST)) was more activated in all simulated cases compared to the lateral one. The most activated component in the hamstring was the biceps femoris long head (BLH), with peak activity of 1.4 BW at the supraphysiological height (Fig 3). Forces in gastrocnemius fascicles increased by 58% and 188% as the landing height increased from 20 cm to 40 cm and 126 cm, respectively.

**Fig 4:**
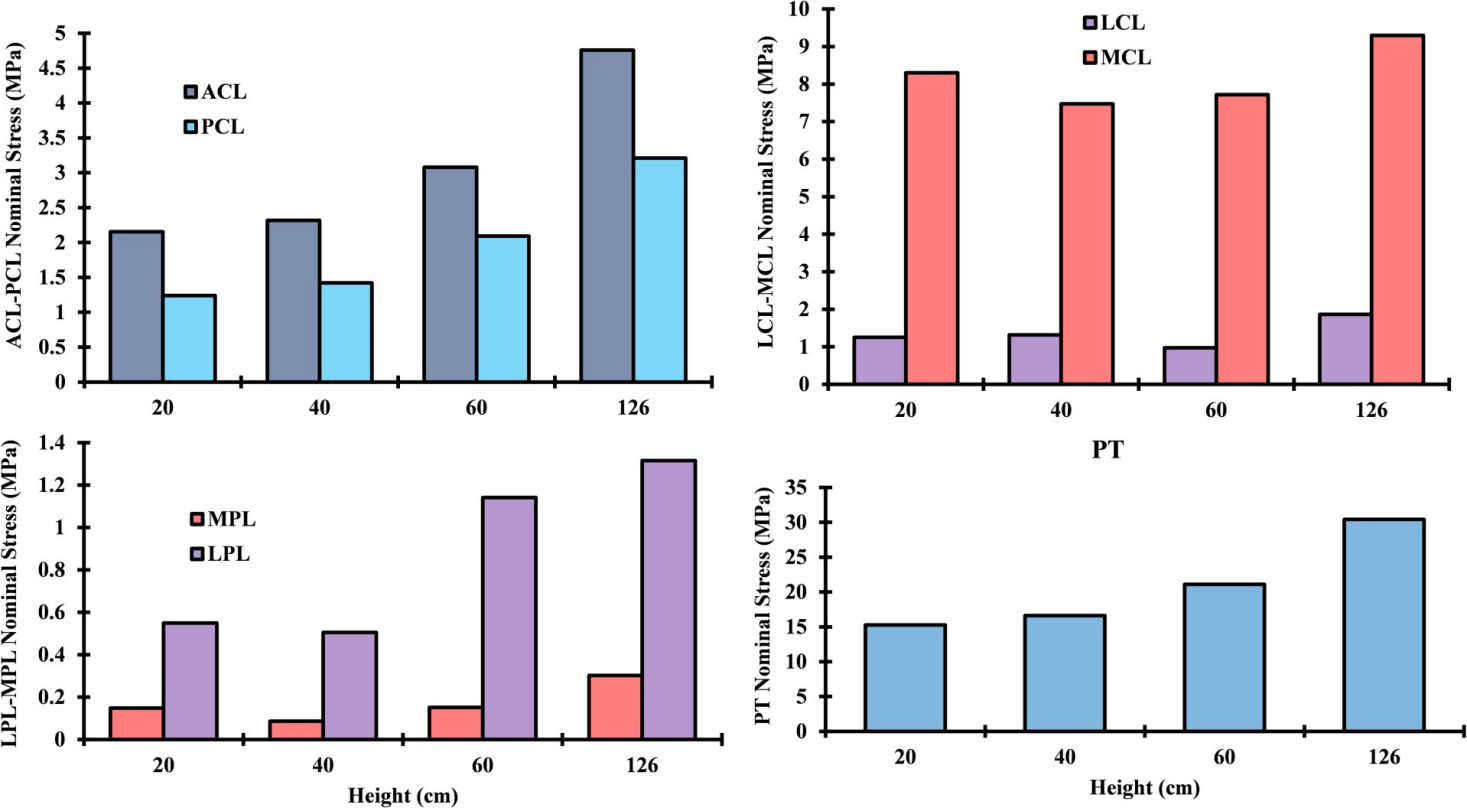
Computed ligament nominal stresses for different drop landing heights at the peak loading instance.

As demonstrated in Fig 4, the loading of the patellar tendon displays a comparable pattern to that of the quadriceps forces, with the maximum nominal stress of 31 MPa being reached at a drop landing height of 126 cm. The anterior cruciate ligament (ACL) and posterior cruciate ligament (PCL) have near nominal stresses throughout the simulated cases, with a slightly higher value on the ACL side. Also, it can be observed that the stresses experienced by the lateral patellofemoral ligament (LPL), medial patellofemoral ligament (MPL), and lateral collateral ligament (LCL) were relatively low compared to the other ligaments. On the other hand, the medial collateral ligament (MCL) was subjected to almost uniform loading across all drop landing heights. In the majority of simulated scenarios, with the exception of the MCL, the load on the ligaments was considerably increased by the impact of drop landing height. Notably, in the case of the ACL, the load more than doubled with a height increase of 106 cm from its base level of 20 cm (Fig 4).

**Fig 5:**
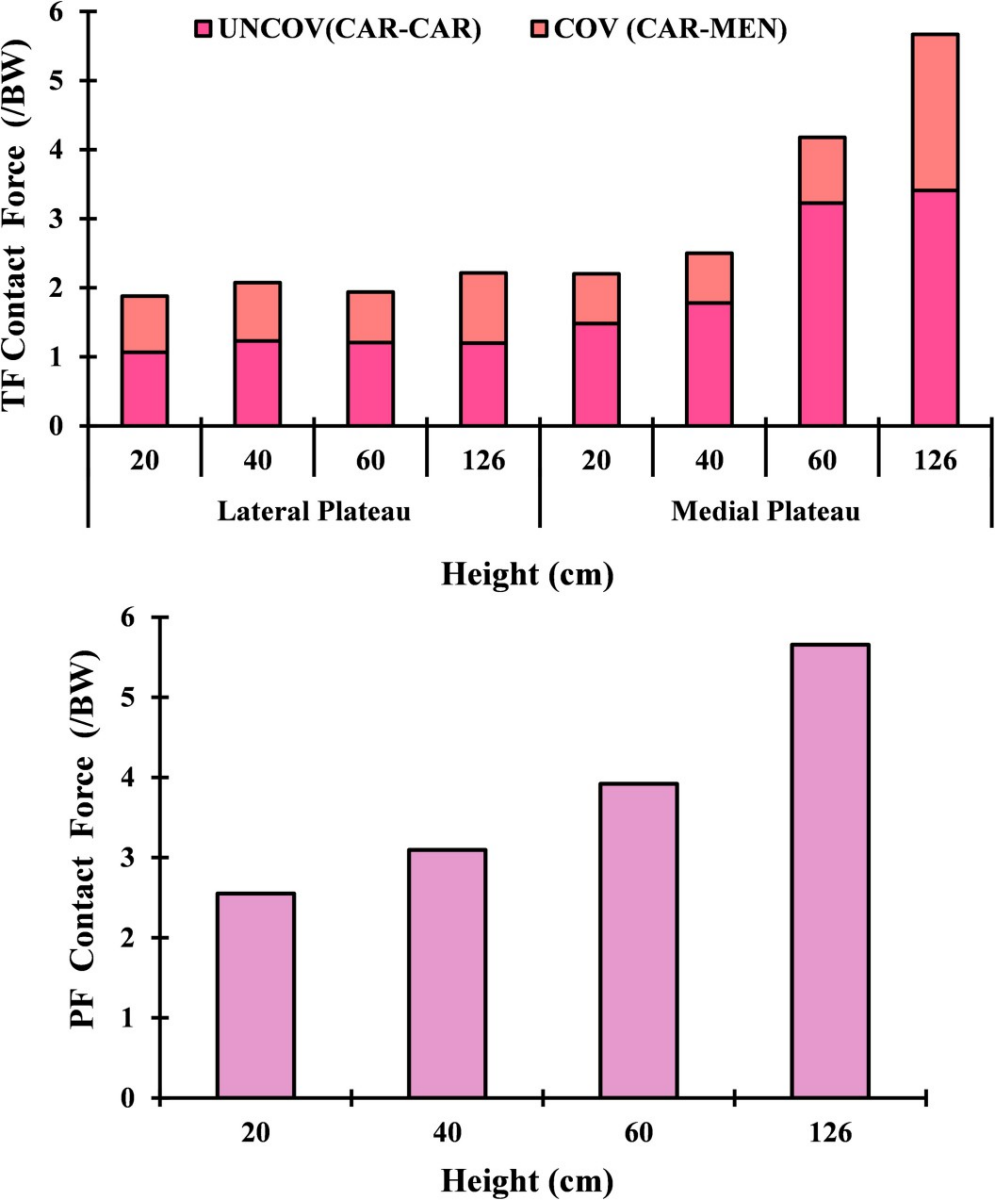
Computed tibiofemoral and patellofemoral contact forces for different drop landing heights at the peak loading instance (cartilage-cartilage (covered) and cartilage-meniscus (uncovered) articular interactions).

**Fig 6:**
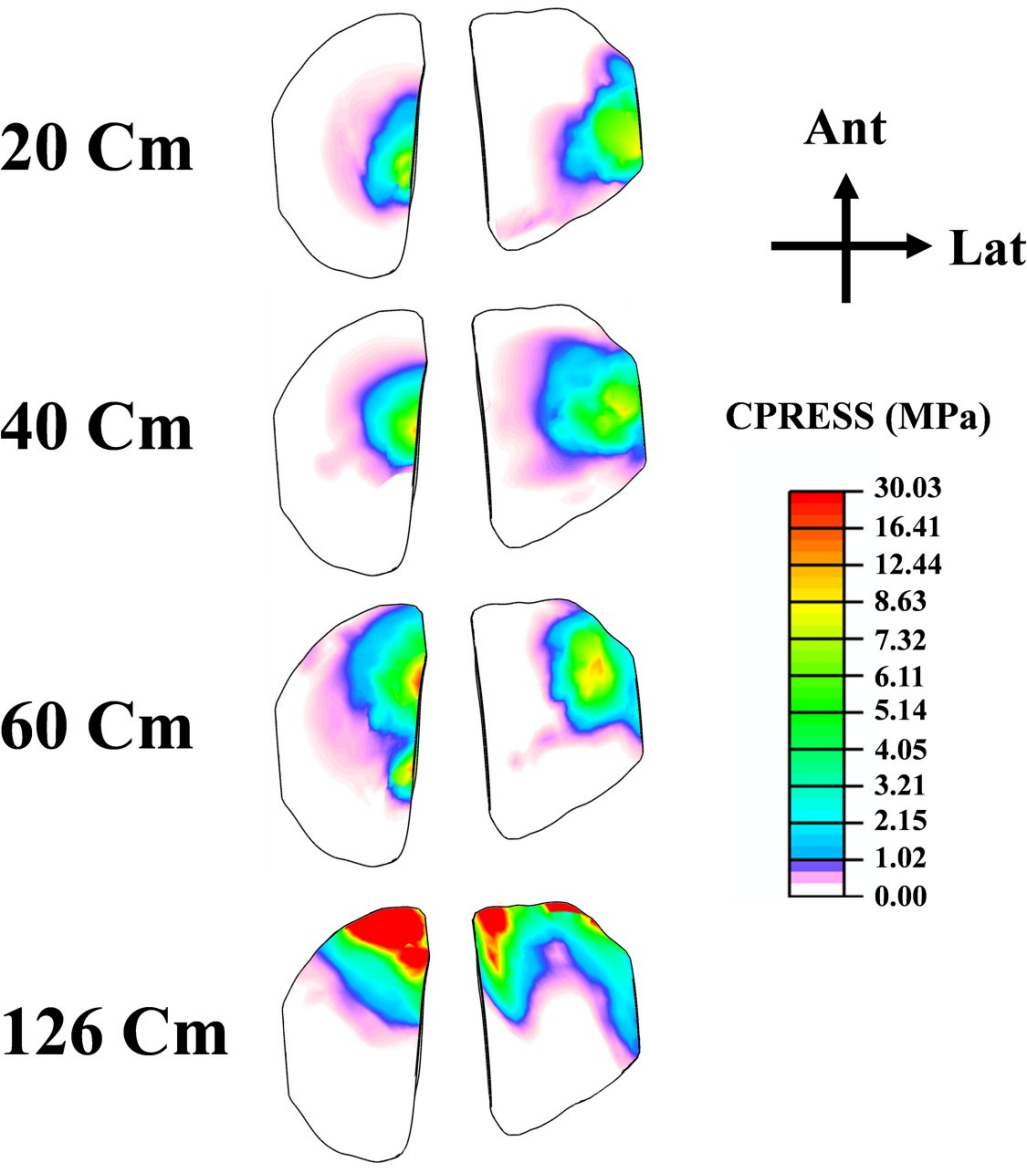
The contact stress experienced by the articular surfaces of the tibial compartments during the peak loading phase at various drop landing heights is presented using a consistent legend to facilitate easier comparisons.

**Fig 7:**
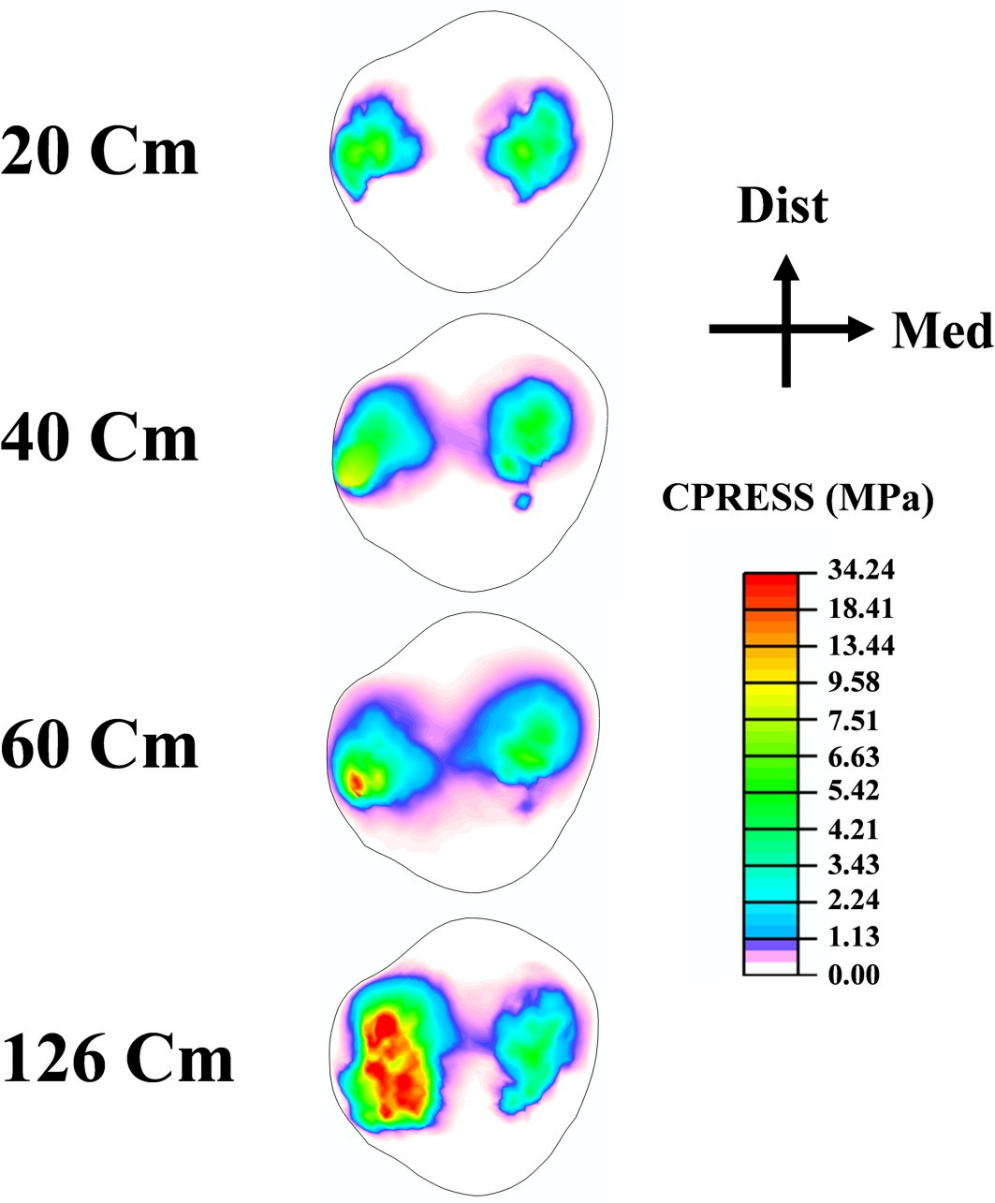
Contact stress at patellar articular surfaces for different drop landing heights at the peak loading instance. Consistent legend is employed to facilitate easier comparisons.

**Fig 8:**
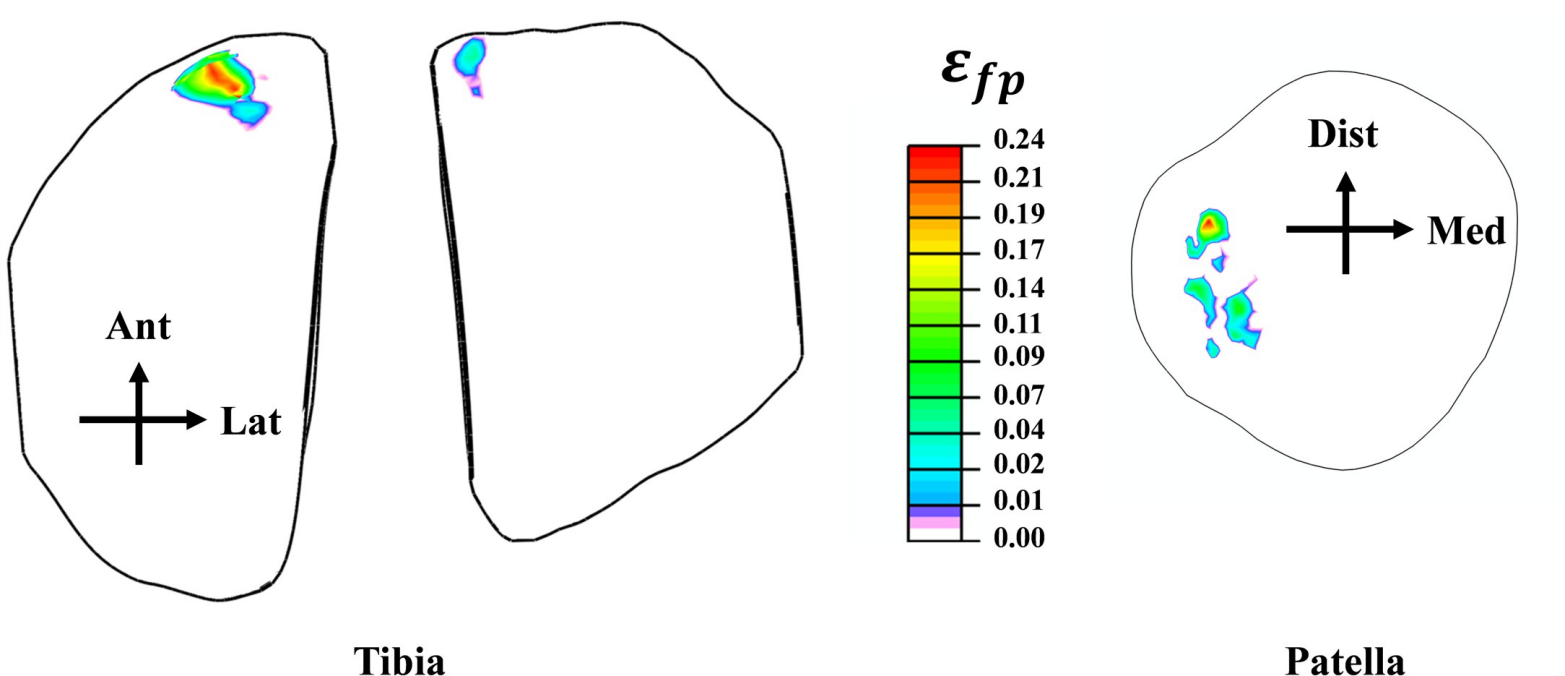
Propagation of cartilage damage distribution (collagen plastic strain) at the supra-physiological loading condition (height = 126 cm).

As a result of the modifications in muscle loading, a substantial tibiofemoral contact force was calculated and transmitted through the tibial compartments via articular interactions between the cartilage-cartilage (covered) and cartilage-meniscus (uncovered) regions (Fig 5). This load peaked at 126 cm of the drop landing height with an uneven distribution amongst the two compartments (lateral and medial), where a higher load has been detected on the medial side after the height of 40 cm. Moreover, the proportion of contact forces transmitted via cartilage was dominant in the tibiofemoral load transfer. Contact pressures (average) were following the same trend as the contact forces. The patellofemoral (PF) joint contact force increased substantially with the increase of the drop landing height (Fig 5). While the patellofemoral and tibiofemoral (TF) contact areas indicated almost a steady state trend after the 40 cm height.TF contact stress distributions were shifted from the middle to the anterior zone and from the lateral to medial compartment after the augmentation of the drop landing height. The peak articular stress of 30.11 MPa was reached at the supraphysiological height (Fig 6). The stress distribution on the PF joint was concentrated more on the lateral side with a maximum value of 34.24 MPa at 126 cm of height (Fig 7). Finally, model prediction indicated that cartilage damage (plastic strain) was initiated at the maximum contact stress location on both TF and PF joints (Fig 8). This damage was near the ACL and LPL footprints in the tibiofemoral and patellofemoral joints.

## Discussion

The focus of this study was to examine the impact of landing height, during the peak loading stage of the drop landing phase, on the mechanical behavior of the knee joint and the failure mechanisms of the articular cartilage. For this purpose, a computational construct connecting a musculoskeletal model to an active/passive knee finite element model was developed. The soft tissue damage models were incorporated into the knee FE model via MD simulation-based collagen mesoscopic model. This computational model was established based on distinct kinematics-kinetics data obtained from healthy individuals (_((((xxx))))_)[62–104]. As far as we know, this is the initial investigation to explore the influence of drop landing height on the active/passive reaction of the knee joint. The results validated our expectations, with muscles and knee soft tissue loads varying considerably with height during the peak loading period of the drop landing phase.

One of the aims of this investigation is to develop a framework that can help identify the factors involved in the mechanisms of cartilage and soft tissue landing injuries. Landing is an essential task involved in many functional and sports activities with the highest prevalence of joint injuries, which even exceed 50% during some activities [99]. Most of these injuries happened at larger landing heights that are often infeasible to measure/determine via laboratory experiments, primarily due to the safety of the human subjects. In addition, the currently adopted analyses cannot detect important variables of interest that govern tissue damage initiation, such as stress and strain. Therefore, regression models were developed during this investigation to overcome the safety barriers to identify the lower limb kinematics/kinetics at the most susceptible point of possible joint injury (peak ground reaction force). To determine the variables of interest (stress and strain), the knee damage model was incorporated with the musculoskeletal model and driven by the kinematics/kinetics regression models. In addition, an iterative simulation technique was implemented to determine the height at which the cartilage damage was initiated. A 1-meter dropping height was considered the initial height with a degraded increment of 20, 10, 5, and 1 cm to identify the critical landing height precisely. As a result, the cartilage damage was detected first at 126 cm, considered the supra-physiological dropping height. Finally, additional analyses have been conducted in this investigation at lower dropping heights to understand better the factors involved in the impact injury mechanism.

Following the apparent increase of the knee flexion moment and rotation with higher dropping height (60 cm to 126 cm) (Fig 2), quadriceps muscle forces increased significantly by an average of 33% (Fig 3). The computed larger load due to the quadriceps being less efficient in generating flexion moments at higher flexion angles observed in the landing case at a higher loading point [96]. Of the middle-quadriceps components, the intermedius muscle experienced the most significant disturbance, with a 58% increase in activity observed. Conversely, the rectus femoris exhibited no alterations with changes in landing height. This could be explained by the inverse polyarticular role of the rectus, which is considered an extensor of the hip and flexor of the knee joints. On the vastus side, the highest alteration has been observed with lateralis components, where the force increased from 1.2 body weight (BW) in the lowest dropping height to 2.8 BW in the supra-physiological dropping height. In addition to the knee flexion moment, the augmentation of vastus lateralis activity may be attributed to its resistance against abduction and internal moments during this particular phase [81].

The medial hamstring muscles, specifically the semitendinosus and semimembranosus, experience significantly higher forces compared to the lateral hamstring muscles (biceps femoris long head and biceps femoris short head) that were associated with the knee varus and hip extension moments. Furthermore, these muscle activations were characterized by a larger activation level of the semimembranosus than the semitendinosus. On the external side of the knee joint, a deferential activation was computed between the biceps femoris components in which the biceps femoris long was much more dominant than the biceps femoris short head. Here, the additional role of the long head in stabilizing the extension/adduction moments of the hip and the constraint imposed on the short head by the knee abduction moment represent a reasonable explanation of the observed activation. The paramountcy of the medial hamstring is thought to play a key role in managing medial knee opening, thereby enhancing overall joint stability to counter the predominant abduction moments [81, 93]. Even with the considerable flexion moments on the knee in all simulated cases, higher activity was computed in the gastrocnemius components, with a larger load on the medial gastrocnemius (MG) components than the lateral gastrocnemius (LG) (Fig 3). The gastrocnemius’ heightened activity was associated with the increased ankle dorsiflexion moment at simulated instances (Fig 2). To counterbalance this antagonistic activity, supplementary forces were calculated in the quadriceps, predominantly borne by the vastus components. Except for the hamstring muscle, greater drop landing heights were usually characterized by larger muscle contraction and cocontraction degrees via superficial electromyography (EMG) measurements [99]. The optimized muscle loads (trends) were evaluated with the previously reported EMG measurements (normalized) [96, 99]. Overall, the computed predictions matched the observed trends in absolute terms. Nonetheless, the estimated muscle forces in the hamstring were consistently greater than the measurements at higher drop landing heights, likely due to the incorporation of deep components in our finite element model. Additionally, the potential errors associated with EMG measurements in larger muscles, along with any attempts to map normalized magnitudes (%EMG) to muscle load, emphasize the need for caution when making such evaluations.

The changes in muscle forces resulting from differences in drop landing height led to relative changes in predicted nominal stresses on the ligaments. These stresses increased simultaneously in the cruciate ligaments (ACL, PCL) with higher balance supported by the ACL. This stress almost doubled at the supra-physiological loading instance. The remarkably higher activity in the quadriceps and hamstring muscles and the accompanying augmentation of the knee flexion rotation may justify the less but unexpected stress observed in the cruciate ligaments, especially in the ACL, and hence did not increase the risk of injury. However, earlier observations reported larger loading on the ACL at a greater height may lead to tissue damage [109]. This latter was not identified at a specific instance of the landing phase, which may lead to the possibility of ligament damage before or after the peak loading point of the phase simulated in our investigation. The details of the knee ligament’s function and damage during landing should be well scrutinized in future studies of landing biomechanics for different landing techniques and heights. The lateral (LPL) and medial (MPL) patellofemoral ligaments were associated with low nominal stresses, which may be attributed to a relatively minor variation in the vastus components’ load distribution. A similar variation of ligament loading has been reported in earlier modeling studies [62, 72].

The current study estimated contact force in both TF and PF joints. In general, the results of this analysis revealed a substantial increase in the tibiofemoral force with the increase of the drop landing height. This augmentation is relative to the considerable rise of the surrounding muscle loads and their related greater joint axial force. Our predicted contact force is in reasonable agreement with previous studies in the literature [96, 99]. At low landing heights, the lateral compartment bears the majority of the load transferred via the knee joint. However, with increased landing height, this contribution shifts more towards the medial side. The segment of the force transferred via menisci is relatively low in all simulated cases, with an average of 39%. Consistent with the alterations in contact loading, an increase in landing height resulted in greater contact stress being experienced by the medial plateau, accompanied by an anterior shift in the center of the contact (Fig 6). The anterior migration of distribution was due to the increased quadriceps activity. This activity has been recognized as the main decelerator during the landing phase, leading to anterior shear force, considered the major contributing factor to anterior tibia translation [68]. Furthermore, a distinct elevation in patellofemoral cartilage stress was computed with the increase in landing height. The stress on this joint is the outcome of the drastic augmentation of the quadriceps and the patellofemoral tendon forces. Our predicted contact stresses were much lower than the earlier computational study of Makinejad et al., [110]. The observed differences in the applied boundary conditions and the properties of the assigned materials to the knee soft tissues can explain this discrepancy in the predicted results. In other words, unlike our proposed model, in their investigation, the absence of a realistic scheme connecting different scales simultaneously from joint kinematics/kinetics to geometry and an accurate anisotropic representation of the tissues’ behaviors may contribute to the substantial increase of the computed cartilage and meniscus stresses [110], since these parameters were considered as an important damper of lower extremity joints loading [4]. Finally, at the supra-physiological loading scenario (landing height = 126 cm), the damage distribution in collagen fibrils started at the anterior-posterior direction and then slightly propagated into the fibrils’ medial-lateral direction in the superficial layer. The damage is eventually expressed in the upper layer of the anterior-medial region of the TF joint. In the PF joint, the damage initiation was also located on the superficial layers on the lateral side near the lateral patellofemoral ligament junction. These results indicated that failure generally originated at the upper layers of the articular cartilage. Identifying these properties (damage initiation and propagation) may inform the interest in biological repair and resurfacing cartilage defects [111–114].

Results in the current work should be considered with a few limiting assumptions. The characterization of the proteoglycan network (matrix) failure was not considered. However, it remains to be seen if including a hybrid model of damage (fibril-matrix) will lead to different mechanical behavior. This study used a single model to represent a population with varying bone structures due to age and gender differences [115–117]. While subject-specific models are considered the gold standard for capturing individual variability in bone structure and material behavior, creating such models can be complicated and time-consuming [118]. It may require access to specialized equipment and data sources such as MRI or CT scans. Therefore, the choice to use a single model was made based on available resources and timeline constraints. It is worth noting, however, that the model used in this study was validated and cross-verified to ensure the ability to produce physiologically acceptable results [18, 24, 25, 119]. While some uncertainty may still exist in its prediction, this process helps create confidence in the model’s accuracy. Co-activity in muscle exertions was not considered. A wide range of ages in regression analyses was considered, which may introduce confounding factors, especially the data from individuals aged 14, still in the stage of skeletal development. However, it is worth noting that the number of studies that included subjects aged 14 was very low, appearing in just 2 of the 42 investigations considered in our analyses [90, 91]. Additionally, these studies analyzed drop landing data collected from female basketball players whose anthropometry was close to the normal female average (F; body mass = 61.9 kg, stature = 173.5 m) [120]. Therefore, their inclusion in the regression model would have had a minor impact on the overall results. Ultimately, the kinematics-kinetics measurements utilized as input data for our model may significantly impact the present findings and conclusions. Even with the high variability of the reported data treating lower extremity kinematics in earlier landing analysis investigations, the wide range of the considered studies during this investigation may generalize the predicted results to a certain extent. Finally, this study also acknowledges the limitation linked to the assigned materials properties of the cartilage matrix from Sajjadenian et al. [39]. This choice was based on the reported equivalence between short-time biphasic and incompressible hyperelastic material responses [4, 121–123], where the high incompressibility level allowed for the utilization of biphasic material properties of the non-fibril parts of the cartilage, specifically for transient response analysis. However, it should be noted that this choice may affect the accuracy of the predicted results, particularly since the osmotic pressure parameters were treated separately from the non-fibril parts of the cartilage in the considered reference [39].

In summary, this investigation provided an engineering framework for identifying the interaction between changes in lower extremity aggregate kinematics and kinetics during drop landing and its associated alterations of knee soft tissues’ basic mechanical behavior. This work allowed us to successfully understand micro cartilage injury due to excessive loading that could lead to acute and focal collapse. If not treated, these focal damages can develop into full-blown clinical OA. Hence, knowing the level of loading that would lead to soft tissue microtrauma can play an important role in limiting the injuries related to the high physical demands of certain services and provides a foundation to explain the increased prevalence of the painful joint disease.

## Supporting information

S1 File (PDF)
