# Supplementary material for: Knee joint biomechanics and cartilage damage prediction during landing: A hybrid MD-FE-musculoskeletal modeling
Source: PLoS One. 2023 Aug 3;18(8):e0287479. doi: 10.1371/journal.pone.0287479 (PMC10399834; doi:10.1371/journal.pone.0287479)
Supplement: S1 File — (PDF) [file pone.0287479.s001.pdf]

Supplementary material

I) Details of the mesoscopic model:

1) Model Structure and Simulation:

In the present work, we use a mesoscopic model of the collagen fibril. This model is built based on the geometry of the atomic scale tropocollagen molecules, which represent the constitutive elements of the fibril. The molecule is formed by a triple helix (two α-1 chains and one α-2 chain), where each chain is composed of a succession of amino acids (glycine, proline, hydroxyproline, hydroxylysine, Arginine, …). A detailed characterization is found in Gauteri et al., [1]. Furthermore, the availability of molecular structure of tropocollagen [2] and the elementary interactions between atoms forming the molecule (C, N, O, H) [3-6] provide a feasible process to model collagen molecules [7-9] via direct (conventional) molecular dynamics (MD) simulations**.** Such simulations are also possible because a single molecule is about 300 nm long, 1.5 nm wide, and contains about 42 thousand atoms, which is a reasonable simulation size for MD.

If it is considered to model the whole fibril, the direct molecular approach is no longer an option: In fact, a typical fibril ranges from 20 nm to a few hundred nanometers in diameter and therefore contains 50 million to a few billion atoms. Unfortunately, modeling and simulating such a fibril is not possible using direct MD because of computational limitations. Therefore, the concept of beads or superatoms in modeling collagen molecules was introduced by Buehler et al. [10]. The coarse-grained approach is based on the concept of abbreviating each set of geometrically adjacent atoms into one superatom (or bead) aggregating together to generate a collagen molecule (fig. 1). These molecules were connected in each proximity via mature (trivalent) and immature (divalent) crosslinks (fig. 1) to generate the fibril. Thus, this approach significantly reduces the simulation's complexity and allows access to sizes otherwise inaccessible by conventional MD.

In this work, we follow the same bead definition by Buehler et al., [11], considering 218 beads per molecule (about ~192 atoms per bead) (fig. 1). Using the exact bead definition is crucial since changing the number of beads will change the characteristics of the interatomic bonds, hence requiring the definition of a new force field. Since the same bead configuration in Buehler et al., [11] was considered, the interatomic potential developed for this specific configuration can be used.

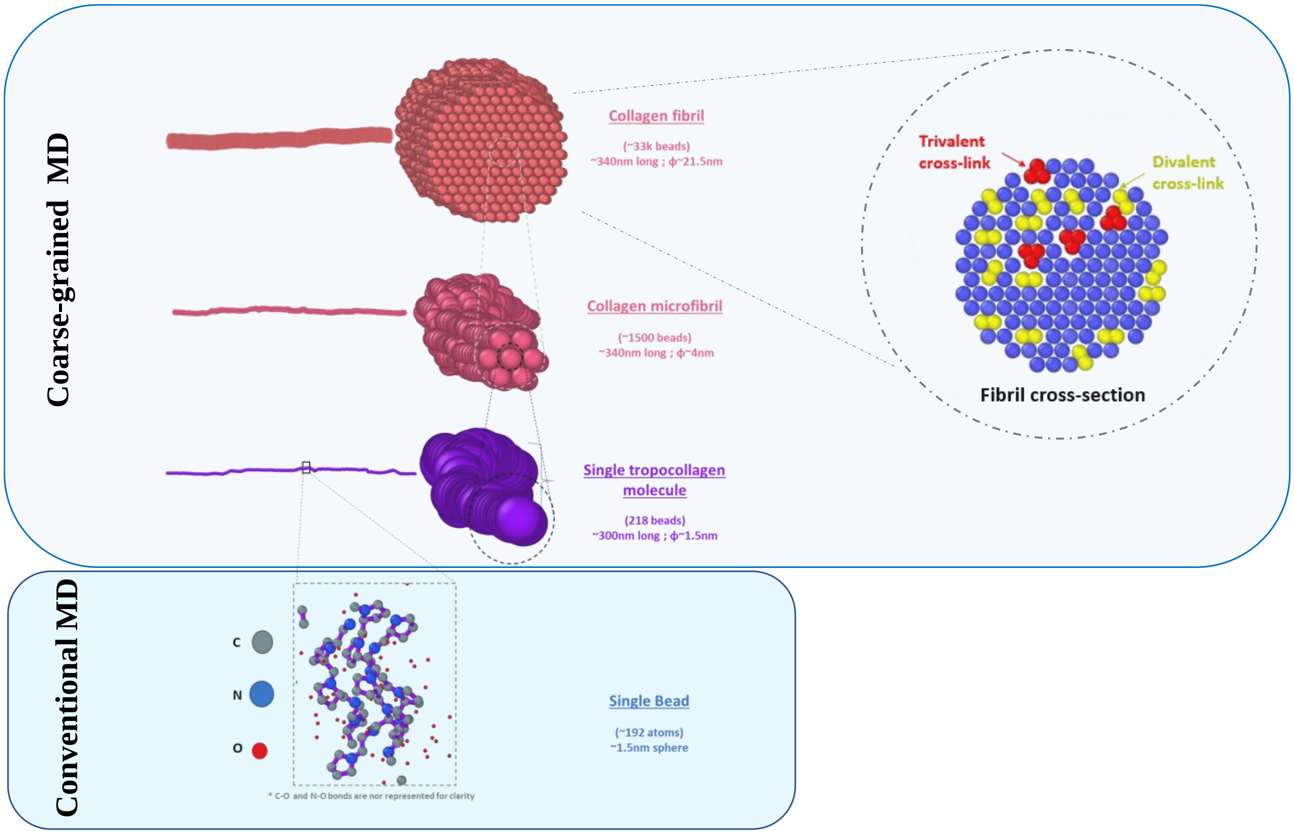

Figure 1: A multiscale representation collagen fibril from its atomic components as well as the cross-section of a collagen fibril. (Beads in yellow represent divalent crosslink and red represent trivalent cross-link).

Starting from the defined molecule, the fibril is then built by replicating the tropocollagen molecule orthogonally to its principal axis. A hexagonal configuration was used with a distance of 16.52Å between molecules, forming a fibril of 21.5nm diameter and containing 151 molecules (32918 Beads). A gap length of 36 nm and an overlap length of 28.2 nm are used (fig. 2). Details on the molecular, mesoscopic model parameters are given in Table 1.

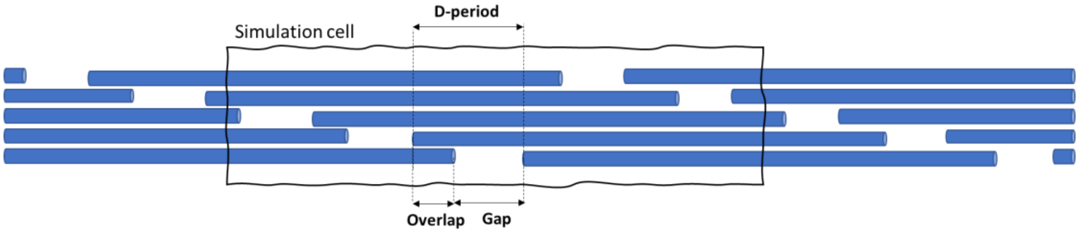

Figure 2: Cross-longitudinal view of the geometrical organization of tropocollagen molecules into the fibril structure.

Table 1: Mesoscopic structural properties

Molecular Properties		Fiber properties	
**Parameter**	**Value**	**Parameter**	**Value**
**Molecule number of atoms**	3134	**Gap [Å]**	400
**Molecule total mass [g/mol]**	287,000	**Overlap [Å]**	~282
**Number of beads per molecule**	218	**D-period [Å]**	~682
**Mass of each bead [g/mol]**	1316	**Length of fibril [Å]**	3410
**Length along principal axis [Å]**	3011	**Hex. lattice constant [Å]**	16.52

The formulation of the coarse-grained model of collagen molecules was implemented in several studies [11-13] and was proven to mimic the actual behavior of the fibril accurately. Three main energies govern the force field.

$$E = E_{inter} + E_{bond} + E_{angle} \qquad (1)$$

The interatomic energy (E_{inter}) is a pairwise Lennard Jones (LJ) interaction between beads from different molecules responsible for keeping the fibril together in the radial direction. The LJ potential is given by:

$$E_{inter} = 4\varepsilon\left(\left(\frac{\sigma}{r}\right)^{12} - \left(\frac{\sigma}{r}\right)^{6}\right) \qquad (2)$$

where σ and ε represent respectively the characteristic distance and the minimum energy of the LJ potential, parameters for pairwise potential are given in Table 3. The bond energy is a hyper-elastic interaction between two adjacent beads from the same molecule. It is represented by three regimes of potential energy that is given by:

$$F_{bond} = \frac{\partial E_{bond}}{\partial r} = \begin{cases} K_{To}(r - r_o) & , r < r_1 \\ K_{T1}(r - \overline{r_1}) & , r_1 < r < r_b \\ 0 & , r > r_b \end{cases} \qquad (3)$$

where K_{To} and $K_{T1}$are spring constants, r_1 is the distance at which the hyper-elastic behavior of the bond is triggered, r_b is the bond-breaking distance, and $\overline{r_1}$ it is a constant calculated to ensure

the continuity of the force field. Parameters for the bond energy are given in Table 2. The angular energy is a harmonic three-body interaction between three adjacent super atoms from the same molecule to control the bending angle between the beads.

$$E_{angle} = K_{\theta}(\theta - \theta_o)^2 \qquad (4)$$

where K_{θ} represents the bending strength, θ_o represents the equilibrium angle, and θ represents the actual angle between the three consecutive beads. Parameters for angle energy are given in Table 3.

Table 2: Bond energy parameters

Parameter	Molecule	Divalent	Trivalent
**r_o - equilibrium distance [å]**	14.00	10.00	8.60
**r_1 - critical hyperplastic distance [å]**	18.20	12.00	12.20
**r_b - bond breaking distance [å]**	21.00	14.68	14.89
**k_{to} - stretching strength constant [kcal/mol]**	17.13	0.20	0.20
**k_{t1} - stretching strength constant [kcal/mol]**	97.66	41.84	54.60

Enzymatic crosslinks are protein-protein bonds that make up most of the crosslinks in collagen fibril. Enzymatic crosslinks are initially formed between telopeptide and helical residues producing immature (divalent) crosslinks connecting the end of the tropocollagen molecule to the nearest neighbor from an adjacent molecule. Then, this immature crosslink may react with another telopeptide residue producing a mature (trivalent) crosslink joining three collagen molecules by connecting the end of the molecule and the two nearest neighbors from adjacent molecules. In this work, hyperelastic behavior is considered for both divalent and trivalent crosslinks (parameters in Table 2). The ratio of trivalent crosslinks to the total number of enzymatic crosslinks is considered to be 33% [14]. Then, we vary the crosslink content to simulate their effect on the mechanical behavior of the fibril. The coefficient β represents the density of molecule ends connected to beads from other molecules (a coefficient β = 100% corresponds to two connected ends per molecule).

Table 3: Pairwise and angle energy parameters

Parameter	Value	Parameter	Value
**ε - Lennard Jones [Kcal/mol]**	6.87	**θ_0 - Equilibrium bending angle [degree]**	164-180
**σ - Lennard Jones [Å]**	14.72	**K_θ - Equilibrium bending constant [Kcal/mol/rad^2]**	14.98

The fibril model was created using MATLAB R2021A by averaging the geometric positions of the atoms in the 3HR2 PDB entry and replicating the molecule in the radial directions. All MD simulations were performed using LAMMPS molecular dynamics software [15]. A 10fs timestep was used. The fibril was relaxed at 300°K for 1 ns using NPT, then NVT to release the residual stress for 1ns. To model the tensile deformation of the fibril, an axial velocity constraint was imposed on both ends of the fibril while keeping the periodic boundary condition in the longitudinal direction to mitigate any surface energy effects. We then use the virial stresses and the fibril volume to compute the stress-strain curves. Finally, the visualization of the results was performed using the OVITO package [16].

2) Model Output

Figure 3(a) shows the stress-strain curve for a single molecule. The curve can be divided into three zones describing three different mechanisms. Zone I where the molecule was stretched along its principal axis and resulting a small stress that was due to the change of the angle energy only. Then zone II with a uniform stretch of the molecule that led to an elastic constant of ~7.9 GPa. Finally, Zone III with a sharp change in strength occurs as the bond distance reaches the hyper-elastic critical distance r_1 (elastic constant is ~47.1GPa). The molecule continues to stretch uniformly until reaching the breaking point. The molecule breaks when interatomic distances reach the breaking distance r_b. Figure 3(b) shows the stress-strain curve for the collagen fibril for different crosslink densities. The overall trend of the curves is consistent with previous work. Increasing crosslink densities increases both the ultimate tensile strength and the ultimate tensile strain of the fibril. When crosslinks are present in the fibril, they provide additional resistance to the shearing between molecules and therefore retard the sliding threshold, increasing the ultimate tensile strain and stress.

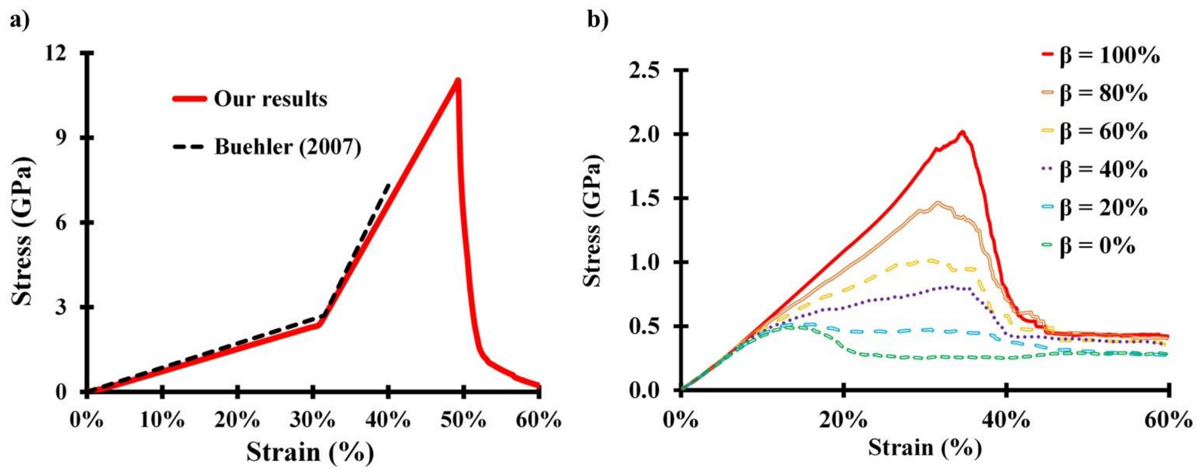

Figure 3: (a) Current computed stress-strain variation in a single TC molecule along with Buehler et al., [11] model predication. (b) Strain-stress plots of the collagen fibril as a function of the amount of crosslink (β) predicted using the coarse-grained model under axial tensile testing.

II) Patellar tendon and ligaments model

The hierarchical composite structure of the ligament is illustrated in figure 4. The tissue is considered as a homogenized continuum in the computations presented herein. Each continuum material point reflects the tissue's statistically homogenous representative volume element (RVE) response. As a result, the macroscopic deformation gradient may be defined as the volume average of the deformation gradient over the RVE under homogeneous or periodic boundary conditions.

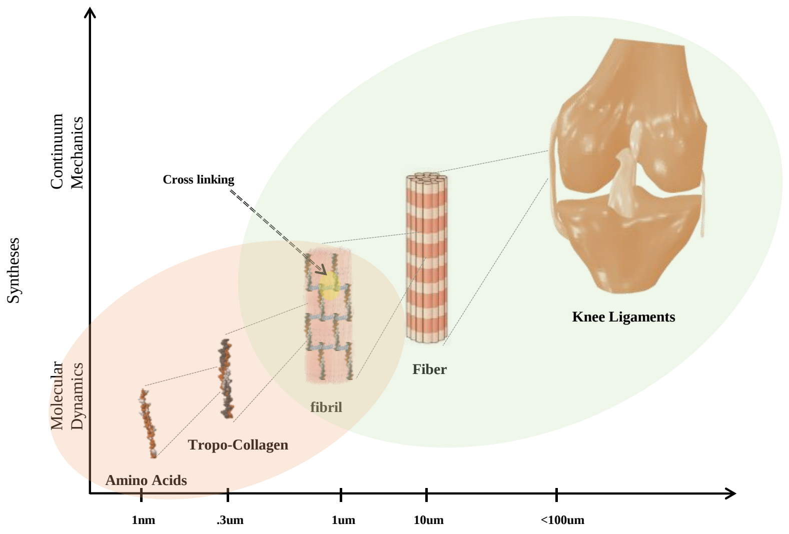

Figure 4: Multiscale hierarchical organization of knee ligaments

1) Fibril Model

The first step is to define the multiplicative decomposition of the deformation gradient to illustrate the relationship between the uniaxial and shear deformation [17,18]. Thus, the total deformation gradient tensor $\overline{F} = \overline{F}_s\overline{F}_f$, where s and f stand for shear and uniaxial deformation. The plastic flow is connected only with the uniaxial deformation (the direction of the fibril) and the total deformation gradient tensor of fibril is $\overline{F}_f = \overline{F}_{fe}\overline{F}_{fp}$, where e and p stand for elastic and plastic components, respectively. The combination of the forgoing two multiplicative decompositions lead to $\overline{F} = \overline{F}_s\,\overline{F}_{fe}\overline{F}_{fp} = \overline{F}_e\overline{F}_p$ where, $\overline{F}_e = \overline{F}_s\overline{F}_{fe}$ (fig. 5):

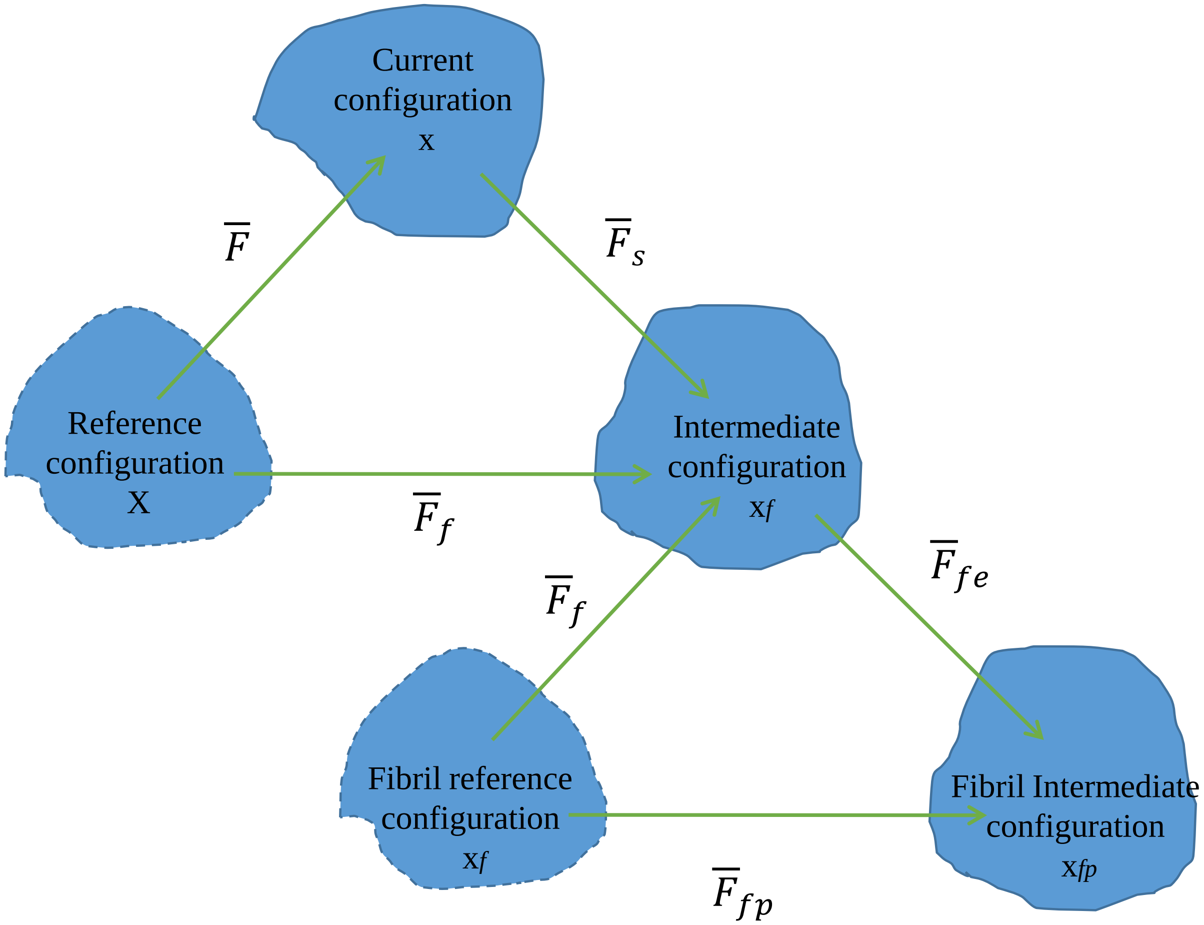

Figure 5: Multiplicative decomposition of the macroscopic deformation gradient of tissue.

This decomposition approach yields a general expression of the strain energy function at the fibril level in the following form:

$$\psi_{fb}(\bar{I}_{1e},\bar{I}_{4e}) = \frac{1}{2}\mu^{fl}(\bar{I}_{4e})(\bar{I}_{1e} - 3) \qquad (5)$$

where the shear moduli μ^{fl} is considered as a function of the elastic fibril deformation with

$$\mu^{fl}(\bar{I}_{4e}) = \mu_o\left(tanh\left(a_1(\bar{I}_{4e}-1)\right) + a_2 exp\left(a_3(\bar{I}_{4e}-I_o)\right)\right) \qquad (6)$$

The hyperbolic structure of the strain energy function is beneficial in fitting the stiffness evolution of the fibril predicted by MD simulation during molecular analyses [19-22]. The plastic flow in the fibril was controlled by the effective stress as follow

$$\Sigma_{eff} = M\colon dev(n_o \otimes n_o) \qquad (7)$$

where M is the Mandel stress

$$M = F^{eT}\bar{\sigma}\,F^{e-T} = F^{eT}\left(2I_{4e}\frac{\partial\psi_{fb}}{\partial I_{4e}}\,n_e \otimes n_e\right)F^{e-T} \qquad (8)$$

where $\bar{\sigma}$ and n_e stand for the macroscopic Kirchhoff stress and the current fibril direction, respectively. By combing equations (7) and (8), it can be easily shown that effective stress takes the form as follows,

$$\Sigma_{eff} = \frac{4}{3}I_{4e}\frac{\partial\psi_{fl}}{\partial I_{4e}} \qquad (9)$$

The following equation gives the flow resistance [23,24].

$$\dot{\Phi} = h\dot{\gamma}\,(1-\frac{\Phi}{\Phi_s}) \qquad (10)$$

where h, Φ, and Φ_s are the softening or hardening rate, yield strength, and saturated flow strength of the fibril, respectively. Owing to fibril softening related to the breakage of crosslinks, the saturated flow strength, Φ_s, is generally chosen to be smaller than the initial yield strength of the fibril (Φ_0) [24]. The latter is a function of the crosslink's density (β) and is defined here by the density function derived from our mesoscopic model (fig. 6). Solving the equality between the yield strength of the fibril (Φ) and the effective stress, lead to a critical value of $\bar{I}_4$ (when $\bar{I}_4 = \bar{I}_{cr}$) that informing the yielding initiation.

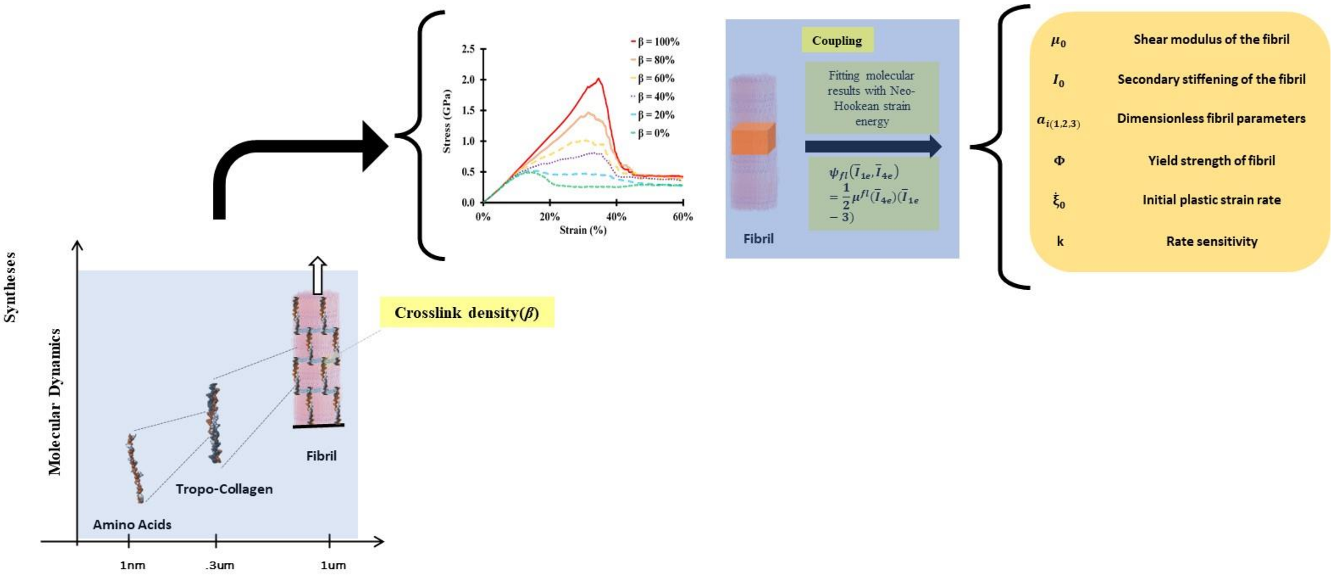

Figure 6: Diagram of coupling between molecular and continuum level mechanics

After that, the single crystal plasticity model was employed to drive the evolution of the plastic strain in the fibril [23-25]:

$$\dot{\xi} = \dot{\xi}_0 \left|\frac{\Sigma_{eff}}{\Phi}\right|^{1/k} sig(\Sigma_{eff}) \qquad (11)$$

where $\dot{\xi}$ is the plastic strain rate, and Φ is the yield strength of the fibril. Here the Karush-Kuhn-Tucker loading/unloading conditions of the system was used as follow

$$\dot{\xi} \geq 0;\; f = (\Sigma_{eff} - \Phi) \leq 0;\; f\dot{\xi} = 0 \qquad (12)$$

After the calculation of the plastic strain rate, the plastic deformation tensor was integrated using the plastic velocity gradient formula [23]

$$\dot{F_p} = \dot{\xi}\, dev(n_0 \otimes n_0)\, F_p \qquad (13)$$

Then, using the deformation gradient decomposition equation, the elastic gradient tensor of deformation was updated as follows:

$$F_e = F\, \mathrm{F}_P^{-1} \qquad (14)$$

Fibrils' plastic stretch was calculated from the basic definition of stretch,

$$\lambda_{fp} = \sqrt{\bar{I}_{4\mathrm{p}}} \qquad (15)$$

where λ_{fp} is assumed to be unity throughout the elastic deformation ($F_p = I\ and\ F_e = F$). Then, the yield distribution and its beginning were identified when the parameter (λ_{fp}) become greater than one.

2) Fiber model

The collagen fiber is modeled as fibril reinforced composite material (fig. 7) with incompressible neo-Hookean matrix characterized by the strain energy function

$$\psi_{fm} = \frac{\mu_{fm}}{2}\left(\bar{I}_{1f} - 3\right) \qquad (16)$$

where μ_{fm} is the shear modulus of the fiber matrix material. The elastic strain energy of the fiber under extension is given by

$$\psi_{fbt}\left(\bar{I}_4, \bar{I}_{4e}\right) = v_{fl}\psi_{fl}\left(\bar{I}_{1ef}, \bar{I}_{4e}\right) + v_{ml}\,\psi_{fm}\left(\bar{I}_{1f}\right) \qquad (17)$$

where $\bar{I}_{1f} = \bar{I}_4 + 2\bar{I}_4^{\ -1/2}$, v_{fl} is the fibril volume fraction of the fiber, and v_{ml} is the volume fraction of the matrix material of the fiber. Then the strain energy function characterizing the fiber under shear is given by

$$\psi_{fs}\left(\bar{I}_{1f}, \bar{I}_4, \bar{I}_{4e}\right) = \frac{1}{2}\mu^{effforb}\left(\bar{I}_{4e}\right)\left(\bar{I}_{1fb} - \bar{I}_{1f}\right) \qquad (18)$$

where $\bar{I}_{1fb} = \bar{I}_1\left(F_f\right) = tr(F^T F)_{fiber}$ and

$$\mu^{efffb}\left(\bar{I}_{4e}\right) = \mu_{fm}\frac{\left(1 + v_{fl}\right)\mu^{fl}\left(\bar{I}_{4e}\right) + \ \mu_0\left(1 - v_{fl}\right)}{\left(1 - v_{fl}\right)\mu^{fl}\left(\bar{I}_{4e}\right) + \mu_0\left(1 + v_{fl}\right)} \qquad (19)$$

represent the shear modulus characterizing the effects of shear interactions at the junction of the fibril and matrix [26-28]. The total strain energy density of the fiber is therefore written as

$$\psi_{fb}\left(\bar{I}_{1fb}, \bar{I}_4, \bar{I}_{4e}\right) = \psi_{fbt}\left(\bar{I}_4, \bar{I}_{4e}\right) + \psi_{fs}\left(\bar{I}_{1fb}, \bar{I}_4, \bar{I}_{4e}\right) \qquad (20)$$

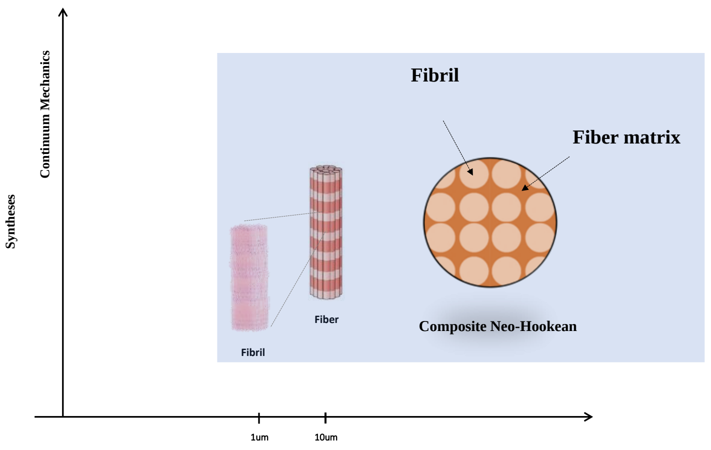

Figure 7: Diagram of coupling between the fibril and fiber level

3) Tissue model

The former process (mixed formulation, fig. 8) is used to treat the tissue as fiber reinforced composite material (fig. 9), where the elastic strain energy of the tissue matrix was considered as follows

$$\psi_m = \frac{\mu_m}{2}\left(\bar{I}_1 - 3\right) \quad (21)$$

where μ_m is the shear modulus of the tissue matrix material. The elastic strain energy of the tissue under extension is given by

$$\psi_{tt}\left(\bar{I}_1, \bar{I}_{1fb}, \bar{I}_4, \bar{I}_{4e}\right) = v_f \psi_{fb}\left(\bar{I}_{1fb}, \bar{I}_4, \bar{I}_{4e}\right) + v_m\, \psi_m\left(\bar{I}_1\right) \quad (22)$$

where v_m *and* v_f are the matrix and fiber volume fraction, respectively, and $v_m = 1 - v_f$. Then the strain energy function characterizing the tissue under shear is given by

$$\psi_{ts}\left(\bar{I}_{1f}, \bar{I}_4, \bar{I}_{4e}\right) = \frac{1}{2}\mu^{eff}\left(\bar{I}_{4e}\right)\left(\bar{I}_1 - \bar{I}_{1fb}\right) \quad (23)$$

where

$$\mu^{eff}\left(\bar{I}_{4e}\right) = \mu_m \frac{\left(1+v_f\right)\mu^{efffb}\left(\bar{I}_{4e}\right) + \mu_m\left(1-v_f\right)}{\left(1-v_f\right)\mu^{efffb}\left(\bar{I}_{4e}\right) + \mu_m\left(1+v_f\right)} \quad (24)$$

The total strain energy of tissue is defined by

$$\psi_t\left(\bar{I}_{1fb}, \bar{I}_4, \bar{I}_{4e}\right) = \psi_{tt}\left(\bar{I}_4, \bar{I}_{4e}\right) + \psi_{ts}\left(\bar{I}_{1fb}, \bar{I}_4, \bar{I}_{4e}\right) + \psi_{vol}\left(\bar{J}\right) \quad (25)$$

We can further write the strain-energy function of the tissue as

$$\psi_t(\bar{I}_1, \bar{I}_{1f}, \bar{I}_4, \bar{I}_{4e}) = \frac{1}{2}(v_f v_{ml}\, \mu_{fm} + v_m\, \mu_m)\left(\bar{I}_4 + \frac{2}{\sqrt{\bar{I}_4}} - 3\right) + \frac{1}{2}(v_f v_{fl}\, \mu_{fl})\left(\bar{I}_{4e} + \frac{2}{\sqrt{\bar{I}_{4e}}} - 3\right) + \frac{1}{2}\mu^{eff}\left(\bar{I}_1 - \bar{I}_1(F_f)\right) + \frac{\mathrm{E_K}}{2}(\bar{J} - 1)^2 \qquad (26)$$

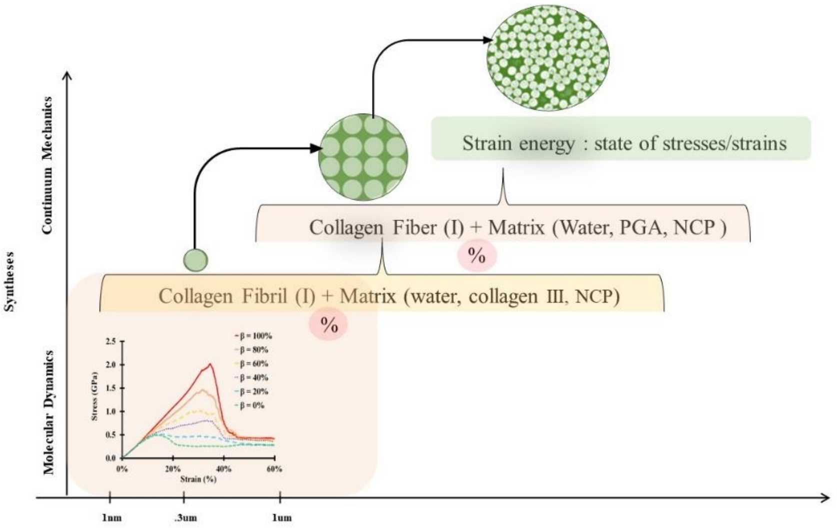

Figure 8: Multiscale mixed formulation used to define ligament as fibril reinforced composite material.

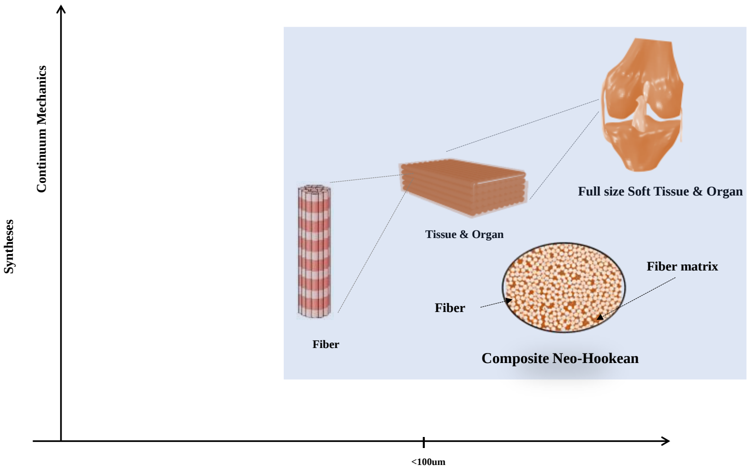

Figure 9: Diagram of coupling between the fiber and tissue level

4) Total stress:

The Clausius-Duhem dissipation inequality at the macroscopic continuum level and the constraint of the incompressibility of soft tissue are given by

$$\begin{cases} \bar{\sigma} : \bar{D} - \dot{\psi}_t \geq 0 \\ \dot{\bar{J}} = J\bar{C}^{-1} : \dot{\bar{C}} = 0 \end{cases} \qquad (27)$$

where $\overline{\sigma}$ is the macroscopic Kirchhoff stress of the continuum tissue and $\overline{D}$ is the macroscopic rate of deformation, and $\bar{J} = \det(\overline{C})$. By satisfying the inequality and the constraint condition, the total Cauchy stress Σ_t can be expressed with fibrillar Σ_f and nonfibrillar Σ_{nf} stress tensors as follow

$$\begin{cases} \Sigma_t = \Sigma_{nf} + \Sigma_f \\ \Sigma_{nf} = \frac{2}{J}\left(\bar{I}_1 \frac{\partial \psi_t}{\partial \bar{I}_1} dev\left(\overline{B}\right) + (E_k \bar{J}(\bar{J} - 1))I\right) \\ \Sigma_f = \frac{2}{J}\left(\bar{I}_4 \frac{\partial \psi_t}{\partial \bar{I}_4} dev(n \otimes n) + \bar{I}_{4e} \frac{\partial \psi_t}{\partial \bar{I}_{4e}} dev(n_e \otimes n_e)\right) & if\, \bar{I}_4 > 1 \\ \Sigma_f = 0 & if\, \bar{I}_4 \le 1 \end{cases} \quad (28)$$

For additional details on the construction of the constitutive model, please refer to the work of [10,11,24,29]. This model was then incorporated into the soft tissue using Vumat-Abaqus (Quasi-static Analysis with Abaqus/Explicit). One continuum element was used for both the fibrillar and nonfibrillar elements of the solid matrix. The proposed material model's basic features and numerical performance were successfully tested with one element and 2080 elements (fig. 10).

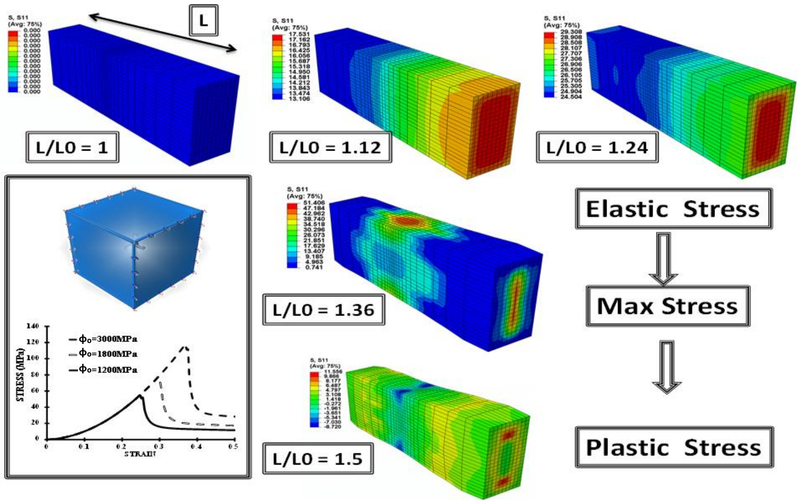

Figure 10: Axial stress distribution under axial displacement with 2080 elements (C3D8R). The framed result (one element) was the axial stress variation when the yield strength of the fibril varied from 1200 MPa to 3000 MPa. This is a numerical verification of the model (VUMAT _ABAQUS), in which we were trying to test the maximum capacity of the proposed model in case of extreme deformation with a random set of materials parameters in the one element and multiple elements cases.

Finally, all materials parameters, multiplicative decomposition, and invariants are listed in the table below.

Table 4: Model parameters.

	**Materials parameters**
μ_m	Shear Modulus of the Solid matrix
$\mathrm{E_K}$	Bulk modulus of the ligament
v_f	volume fraction of the fiber
$\mathrm{n}_{0(1,2,3)}$	fibril direction in the reference configuration
$\mathrm{n}_{(1,2,3)}$	fibril direction in the deformed configuration
$\mathrm{n}_{\mathbf{e}(1,2,3)}$	fibril direction in the deformed configuration associated with the elasto-plastic flow
μ^{efffb}	The effective shear modulus involves the effects of shear interactions at the interface of the fibril and fiber matrix.
μ^{eff}	The effective shear modulus involves the effects of shear interactions at the interface of the fiber and tissue matrix.
μ_{fm}	Shear modulus of the fiber matrix
μ_0	Shear modulus of the fibril
v_{fl}	Volume fraction of the fibril
I_0	Secondary stiffening of the fibril
$a_{\mathrm{i}(1,2,3)}$	Dimensionless fibril parameters
Φ_0	Yield strength of fibril
$\dot{\xi}_0$	Initial plastic strain rate
k	Rate sensitivity
h	Hardening rate
**Multiplicative decomposition and invariant**	
$\overline{\mathrm{F}} = \overline{F}_e \overline{F}_f^p$	The elasto-plastic multiplicative decomposition of the deformation gradient
$\overline{I}_1 = \mathrm{tr}(\overline{\mathrm{F}}\,\overline{F}^T)$	First invariant
$\overline{I}_4 = n_0^t \overline{\mathrm{B}}\, n_0$	Invariant related to the fibril stretch

$\bar{I}_{1f} = \bar{I}_4 + 2\bar{I}_4^{\,-1/2}$	First invariant of fibril
$\lambda_f = \sqrt{\bar{I}_4}$	fibril principal stretch

5) Materials calibration:

Considering the above-proposed model, 12 parameters (unknowns) determine the knee connective tissues' micro and macro mechanical behavior, and most of them are related to the fibril and fiber properties. These parameters are divided into two sets, one treating the fibril response covering eight parameters (μ_0, $I_0, a_{\mathrm{i}(1,2,3)}, \Phi, \dot{\xi}_0, k$) and another for the rest covering four parameters (μ_m, μ_{fm}, v_f , v_{fb}). However, these four parameters were fixed based on our previous calibration process [30], where a statistical calibration is followed to find the unknown probability distribution function (PDF) (fig. 11).

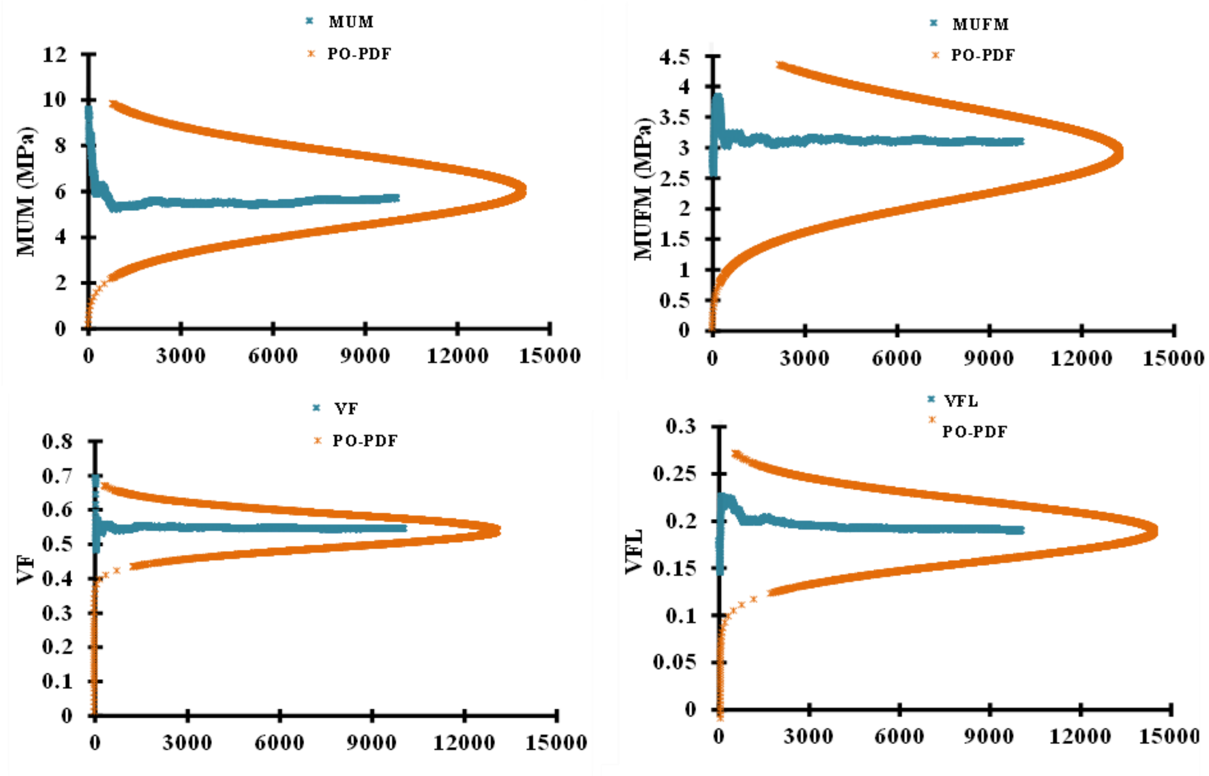

Figure 11: Example of ACL Posterior probability distribution function (PO-PDF) and mean of calibrated material parameters (Bayesian calibration with 10000 MCMC). μ_m (MUM) Shear modulus of the ligament matrix, μ_{fm} (MUFM) Shear modulus of the fiber matrix, v_f the volume

fraction of the fiber, v_{fl} the volume fraction of the fibril, please see Adouni et al., [30] for more details.

The fibril parameters were calibrated to strictly map the MD simulation results at different cross links levels using a nonlinear optimization function from Matlab (*lsqnonlin*) which minimized an objective function *f(x)* (Eq. 29) in a least-square sense ($\sum_n f(x)^2$)). The optimal objective function compromises the stress predicted via molecular dynamics and the finite element model.

$$f(x) = \frac{\sigma_{fl}^{FE}(x) - \sigma_{fl}^{MDS}}{\sigma_{fl}^{MDS}} \quad (29)$$

The initial inputs of the eight fibril parameters were selected based on a plausible range of values using the Latin hypercube sampling MATLAB function. Then these initial inputs were iterated via the optimization procedure to ensure an unbiased estimate of the fibril parameters. Furthermore, as an additional step of verification, the minimum set of outcomes of the optimization process were then used as an input to *globalserch* solver (gs) to ensure the best fit used to drive the fibril hyperelastic model employed in the subsequent FE simulations. As a outcome, the coefficient of determination R^2 were found equal to 0.938 ± 0.06. Thus, the fitted curves exhibit acceptable fits to the MDS data for the different level of cross links. The different cross link density of the patellar tendon and ligaments were selected based on our previous aggregate probabilistic calibration (Adouni et al., [30]). In this selection 20% density were assigned to the patellofemoral ligaments, 60% to ACL, PCL, and LCL, and 80% to the MCL and finally 100% to the patellar tendon.

<u>III) Cartilage Model:</u>

1) Fibril model:

To simulate fibril behavior, a nonlinear constitutive modeling approach developed by Sajjadinia et al., [31] in which the stress of the fibril can be defined as:

$$\begin{cases} \sigma_i^f = \left\{ \frac{\eta_o^s}{J} ln\varepsilon_f \left(E_o\varepsilon_f + E_\varepsilon {\varepsilon_f}^2 \right) (n \otimes n) \right\}_i & \varepsilon_{f_i} \succ 0 \\ \sigma_i^f = 0 & \varepsilon_{f_i} \preccurlyeq 0 \end{cases} \quad (30)$$

Where n and ε_f are the current direction and logarithmic strain of the fibril, respectively. E_o and E_ε are the collagen stiffening coefficients (initial and strain-dependent) and η_o^s is a depth-depend elastic materials parameter. The collagen networks were defined as primary and secondary bundles (i) of fibrils based on their orientation relative to the articular cartilage depth. The fibrils were oriented perpendicular to the subchondral junction and turned gradually in the middle zone to become parallel to the articular surface (fig. 12). The integration of the fibril stress equation with respect to strain in its axial form led to the strain-energy function (W_{fl}) [32]. The increase of this strain energy is unlimited under the strain increase, even though no material can realistically sustain such large strains without any plastic damage or material failure. Inclusion of a damage parameter [33-35] to describe the degeneration of material properties during mechanical loading is limited as the experimental calibration of damage theories are challenging and difficult to measure directly. Therefore, instead of the traditional damage theories, reasonable alternative theories are required to unfold the bulk material failure, which can be simplistically achieved by softening hyperelasticity. The softening hyperelasticity approach for modeling nonlinear materials failure was proposed by Volokh [36-38] and Natli [32] and has been considered in this work to unify the nonlinear elasticity with plastic (failure) descriptions, capturing the softening of the collagen fibril, observed in molecular dynamics simulations. The softening of the fibrils, subjected, was captured by a constant Φ, *energy limiter*, which can be defined as the critical failure energy - the maximum strain energy an infinitesimal volume of material can sustain without failure. However, the energy limiter can be construed as the material toughness analogous to the critical energy release rate in the classical fracture mechanics [39-41]. The limiter automatically induces stress bounds in the constitutive equations. The strain energy function of the fibril is modified with the inclusion of the energy limiter and takes the following form:

$$\psi(\Phi, W_{fl}) = \frac{\Phi}{m}\left\{\Gamma\left(\frac{1}{m}, 0\right) - \Gamma\left(\frac{1}{m}, \frac{W_{fl}^m}{\Phi^m}\right)\right\} \qquad (31)$$

where Γ is the upper incomplete gamma function expressed as $\Gamma(s,x) = \int_x^\infty t^{s-1}\, exp(-t)\, dt$, $W_{fl}$is the strain energy of the intact (without failure) fibril, and the dimensionless material parameter m controls the sharpness of the transition of material softening in the Cauchy stress. Differentiating of the modified strain energy (including chain rule and $\frac{\partial\psi}{\partial W_{fl}} = exp\left(-\frac{W_{fl}^m}{\Phi^m}\right)$,

which is the outcome from the derivative of the upper incomplete gamma function shown as, $\frac{\partial \Gamma(s,x)}{\partial x} = -x^{s-1}\, exp(-x)$ [42]) yields the following fibril stress under uniaxial tension,

$$\sigma_i^{fl} = \sigma_i^f\, exp\left(-\frac{W_{fl}^m}{\Phi^m}\right) \qquad (32)$$

2) Cartilage model:

The articular cartilage was then modeled using incompressible hyperelastic behavior reinforced by the developed continuum-damage model of the fibril. The Cauchy stress (σ^c) in the used model was decomposed into a non-fibrillar (σ^{nf}) and fibrillar (σ_i^{fl}) parts as follow:

$$\begin{cases} \sigma^c = v_f \sigma^{fl} + (1 - v_t)\sigma^{nf} & \\ \sigma^{nf} = \eta_0^s \left[-\frac{lnJ}{6J} G_m \left(\frac{3\eta_0^s\, lnJ}{\eta_0^s - 1} - 3\frac{J + \eta_0^s}{J - \eta_0^s} - 1 \right) I + \frac{G_m}{J}\left(FF^T - J^{2/3} I \right) \right] + \frac{1}{D}(J-1)^2 & \\ \sigma_i^{fl} = \sigma_i^f\, exp\left(-\frac{W_{fl}^m}{\Phi^m}\right) & \varepsilon_{f_i} \succ 0 \\ \sigma_i^f = 0 & \varepsilon_{f_i} \preccurlyeq 0 \end{cases} \qquad (33)$$

Where F and J are the deformation gradient tensor and the volumetric deformation, respectively. G_m is the shear modulus and v_f is the relative collagen fibril volume fraction. For more details on the formulation of the material, please see prior works [31,43].

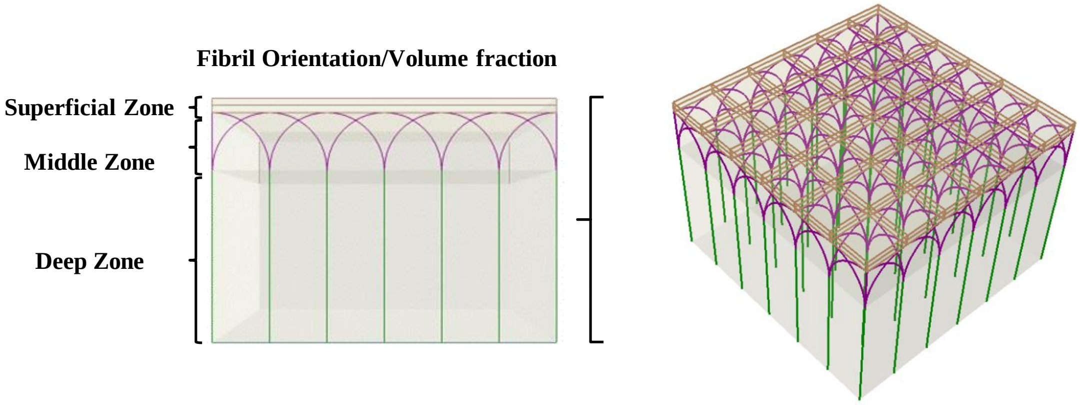

Figure 12: Schematic diagram showing the orientation of the collagen fibrils as a function of the depth in the articular cartilage.

The parameters that are driving the fibrils response of the articular cartilage x_C = (E_0, E, Φ, m) were calculated by fitting them to 70% of the predicted results of MD simulation (fully cross-linked fibril $\beta = 100\%$). This later was set to reflect the fibril (collagen-I) response

in which a different tropocollagen molecule than the cartilage (collagen-II) was employed. Despite this observed difference between the tropocollagen molecules forming cartilage and ligament, the rest of fibril synthesis is almost the same [44]. However, experimental and theoretical investigations [45-47] have shown a higher stiffness (~30%) of the heterotrimeric fibril (collagen I) compared with homotrimeric fibril (collagen II) with almost the same trends of nonlinearity. This is due to the lower cross-link density between the homotrimeric tropocollagen molecules compared with the hetero [48]. The same fitting procedures used to determine the ligaments' behavior were used for the articular cartilage, representing coupling between the molecular and continuum syntheses. The rest of the parameters were fixed based on the earlier investigation of Sajjadinia et al., [31]. This model was then incorporated into the cartilage structure using VUMAT-Abaqus (Quasi-static Analysis with Abaqus/Explicit). The fibril damage mechanism was implemented via the state variable function (STATV) available within the material subroutine Abaqus-VUMAT, the validity of this choice was based on the continuity of the applied load during the landing simulated cases.

IV) Knee Model:

1) Geometry and meshing:

An anatomically accurate model of knee joint consisting of the femur, tibial and patellar bones, articular surfaces, as well as the origins and insertions of the ligaments, was derived from digitized magnetic resonance image (MRI) transverse contours (OpenKnee public domain repository at Simtk.org). The knee specimen (female subject: Age 70 years; Height =170 cm; Weight =77 kg) was scanned at Cleveland Clinic (Biomechanics laboratory) using a one Tesla extremity MRI scanner (Orthone, ONI Medical Systems Inc, Wilmington MA). A scanning protocol that provided a good contrast for the soft tissues within the same scan was used. The protocol characteristics are presented in Table 5.

Table. 5: The magnetic resonance imaging settings (OpenKnee).

Prescan Parameters	Sagittal	Axial	Coronal
Prescan	Auto	Auto	Auto
Center freq.	Peak	Peak	Peak
**Scan Parameters**	**Sagittal**	**Axial**	**Coronal**

Pulse Sequence	GE3D	GE3D	GE3D
FoV	150	150	150
BW	20	20	20
Frequency	260	260	260
Phase	192	192	192
TR	30	30	30
TE	8.9	8.9	8.9
Flip Angle	35	35	35
Time	5.03	3.19	3.30
Echo Train	1	1	1
NEX	1	1	1
**Scan Options**	**Sagittal**	**Axial**	**Coronal**
Graphics SL	Y	Y	Y
Minimum TE	Y	Y	Y
No phase wrap	Y	Y	Y
RF spoiling	Y	Y	Y
Flow comp	N	N	N
Magnetic transfer	N	N	N
Partial data	N	N	N
Inversion recovery	N	N	N
Spatial saturation	N	N	N
Fat Suppression	N	N	N
**Slice Parameters**	**Sagittal**	**Axial**	**Coronal**
Number of slices	70	45	60
Slice thickness (mm)	1.5	1.5	1.5
Gap (mm)	0	0	0
Range (mm)	105	67.5	90

The knee was placed in full extension and the scanning process employed a 3D spoiled gradient-echo sequence with fat suppression, TR = 30, TE = 6.7, Flip Angle = 200, Field of View

(FOV) = 150 mm X 150 mm, Slice Thickness = 1.5 mm. The imaging was conducted in three anatomical planes: (axial, sagittal, and coronal). About 18 minutes was spent finishing the process of scanning. These images are optimal to differentiate between the musculature, tendons, tissue fascia, and bone [49]. The image data set was then imported into an MRI viewing and segmentation analysis package (3D slicer 4.8) and re-sampled in the sagittal, coronal, and axial planes. The muscle-bone junctions were identified from the MRI images following the procedure outlined in Dhaher and Kahn [50]. Polygonal surfaces were used to generate a FE mesh of the knee joint using the Hypermesh (Altair Engineering, Troy, MI) and SOLIDWORKS (CAD) pre-processor. The structure of the tibiofemoral joint was adjusted to match the given dimension in the open knee public domain repository at Simtk.org [49]. Bones were defined as rigid bodies [51] using 4-node quadrilateral elements that injunction with elastic boundaries with the articular cartilages. Eight-node hexahedral elements were used to represent the articular cartilages, ligaments, and menisci (fig. 13).

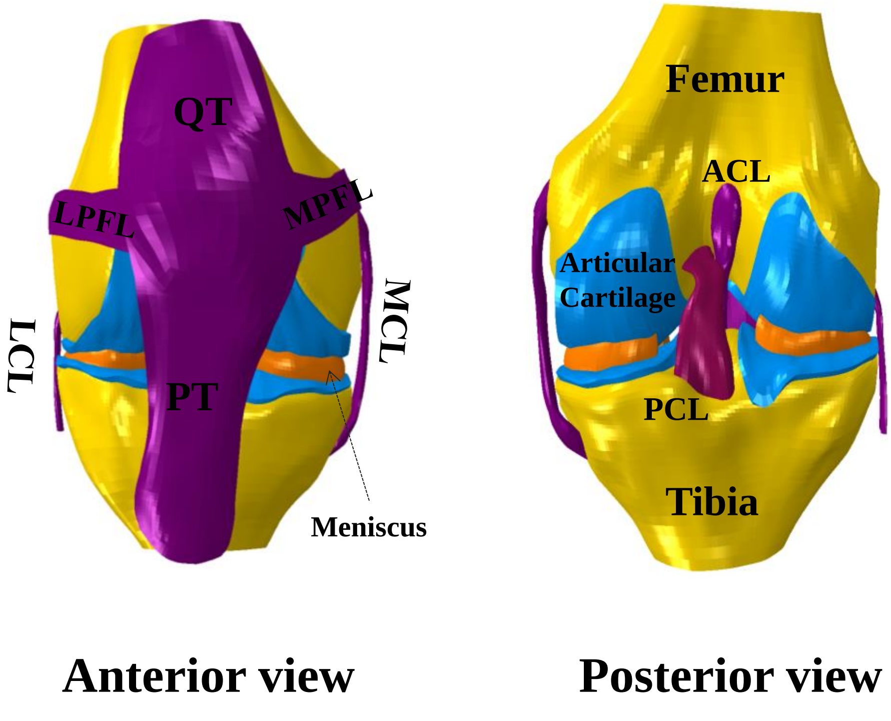

Figure. 13: Anterior and posterior views of the three-dimensional finite element model of the knee showing the corresponding soft tissues and articular surfaces acting on the bones. Anterior cruciate ligaments (ACL), posterior cruciate ligaments (PCL), medial and lateral collateral ligament (MCL, LCL), lateral patellofemoral (LPFL), medial patellofemoral (MPFL), quadriceps

tendon (QT), and patellar tendon (PT) cartilage layers and menisci are shown. More details on the system of axes and the joint center calculations can be found in [49].

The mesh of the model was obtained through a sensitivity analysis, where a maximum of 6% difference in the von-Mises stress was considered (Table. 6).

Table. 6: Mesh of the knee joint.

Set	Number of elements	Types of elements
**Femoral cartilage**	34452	C3D8R
**Tibial cartilage**	17694	C3D8R
**Patellar cartilage**	8736	C3D8R
**Meniscus**	11440	C3D8R
**ACL**	32768	C3D8R
**PCL**	41984	C3D8R
**LCL**	53248	C3D8R
**MCL**	40960	C3D8R
**MPL**	3165	C3D8R
**LPL**	2795	C3D8R
**PT**	32800	C3D8R
**QT**	5117	C3D8R
**Bones (femur, tibia and Patella)**	27560	S4R

2) Fibril orientation

For each C3D8R element, a local coordinate system was used to define fibril orientation and implemented by a python script. This script read the connectivity of each element and defined the local cross-sectional plane (x’, y’) and its normal vector along the local z’-direction (fig. 14).

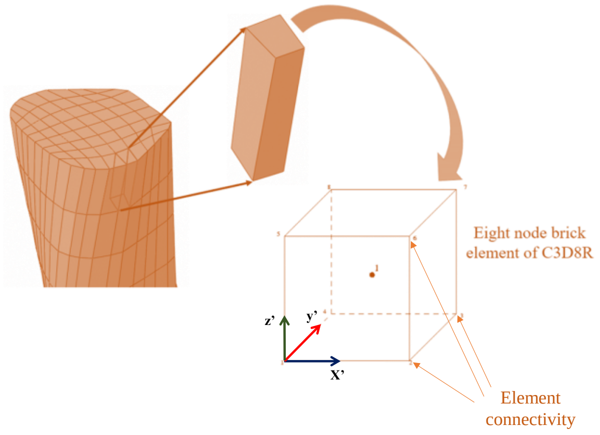

Figure. 14: Element local coordinate system (x', y', z') employed to define the orientation of the fibril.

3) Interaction and loading analyses

The explicit algorithm was used during all the simulations with short step time (0.01s) to mimic quasi-static analysis. The boundary conditions were applied gradually using an exponential function for the normalized amplitude (fig. 15) to attain smooth results. Frictionless interaction property was considered for driving surface-to-surface contact formulation. Computations were performed using an Intel(R) Core (TM) I7-7700@CPU3.60 GHz dual processor, 32.0 GB of RAM, HP EliteDesk machine.

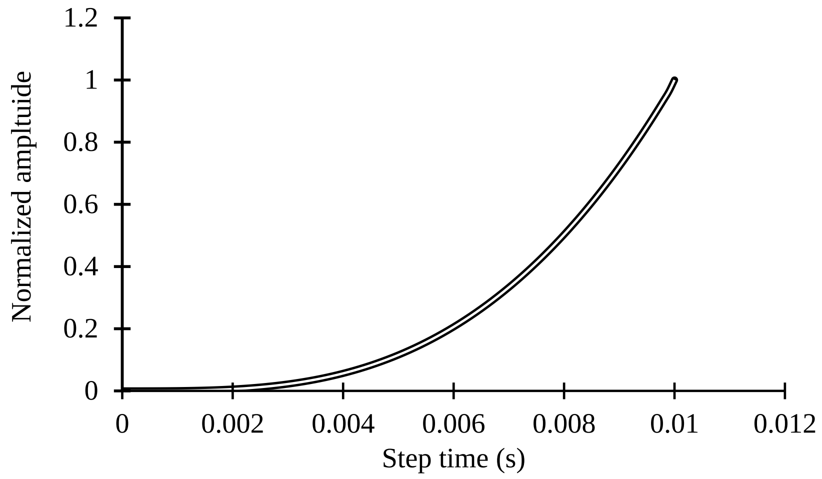

Figure. 15: The normalized amplitude as a function of the step time used to guide the application of boundary conditions during knee modeling.

V) Knee Model Validation and Verification:

A knee axial compressive loading scenario was simulated to test the capability of the implemented multiscale models of the ligaments and articular cartilage within the FE model of the intact joint. The tibial degrees of freedom were free except for the tibial flexion for simulated loading conditions, while the femur was fully constrained. The compression load was applied axially parallel to the tibial bone at full extension and in flexed posture to yields cartilage normal loading at the sub-yielding state and damage at post-yielding state. For the sub-yielding state, simulations were conducted for compression forces ranging from 200 to 2000 N, and this is to facilitate the validation and cross verification with the available experimental data treating tibiofemoral joint interaction reported in the literature [52-55]. Furthermore, axial loads were gradually applied parallel to the tibial axis to trigger cartilage damage at 30° flexion in an attempt to qualitatively verify our predictions of the post-yield response [56].

Axial displacement reached a maximum of 1.47 mm when the knee compression load increased from 0 to 2000 N (fig. 16). The tibial bone moves laterally and anteriorly by 2.6 mm and 1.3 mm, respectively. A high internal rotation was computed (~6°) compared to abduction one (~0.4°). Patellofemoral ligaments (MPL and LPL) remained slack while ligament forces in the tibiofemoral joint under 2000N compressive load reach 104 N, 8 N, 22 N, and 19 N, for ACL, PCL, MCL, LCL, respectively. Almost an even load distribution between tibial plateaus was computed with a difference not exceeding 33 N (supported by the lateral plateau). The Majority of the joint load was transferred via the cartilage-cartilage interaction (uncovered zone) on the lateral plateau (72%), while this percentage decreased by almost 31% on the medial one. The knee contact area followed the same trend of the contact force and reached a maximum of 1418 mm^2 at 2000 N axial load (fig. 17). Tibiofemoral mean contact pressure increased from 0.91 to 1.85 MPa when the applied load increased from 1000 to 2000 N, respectively (fig. 18). A maximum of ~4 MPa was computed as the peak contact pressure on the lateral plateau (fig. 19). The superficial (7.32 MPa) and vertical (6.01 MPa) fibrils were mostly stressed in the structure of the articular cartilage, specifically in the area located under and away from the central loading area, respectively. Low stress has been computed at the random fibril zone. All these computed parameters were in good agreement with the former experimental and modeling investigations[52-55] (fig. 16-19).

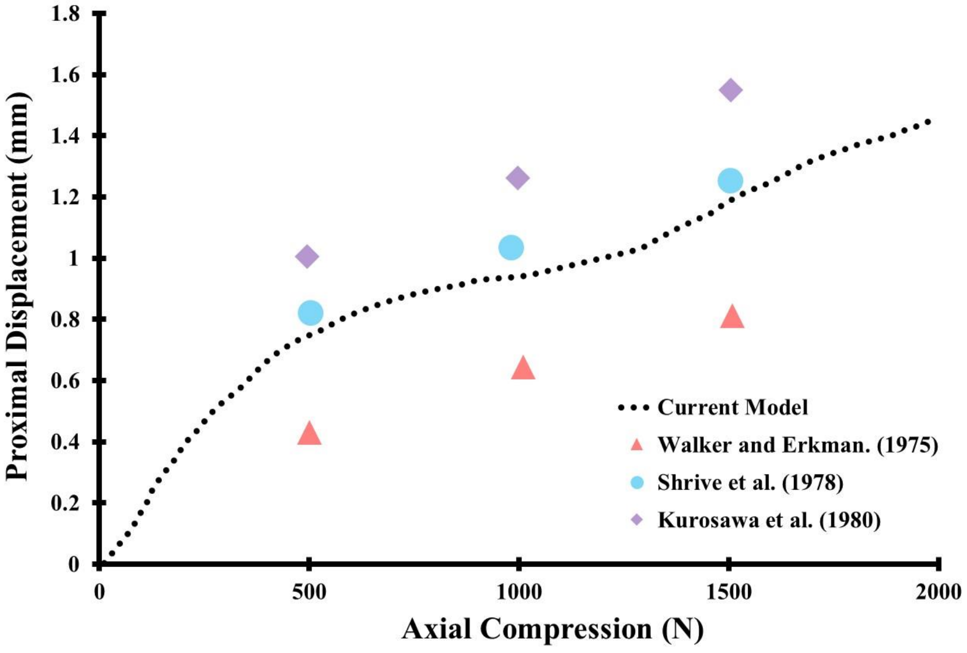

Figure 16: Tibial proximal displacement under axial compressive load up to 2000 N, experimental measurements under almost the same boundary conditions shown for comparison.

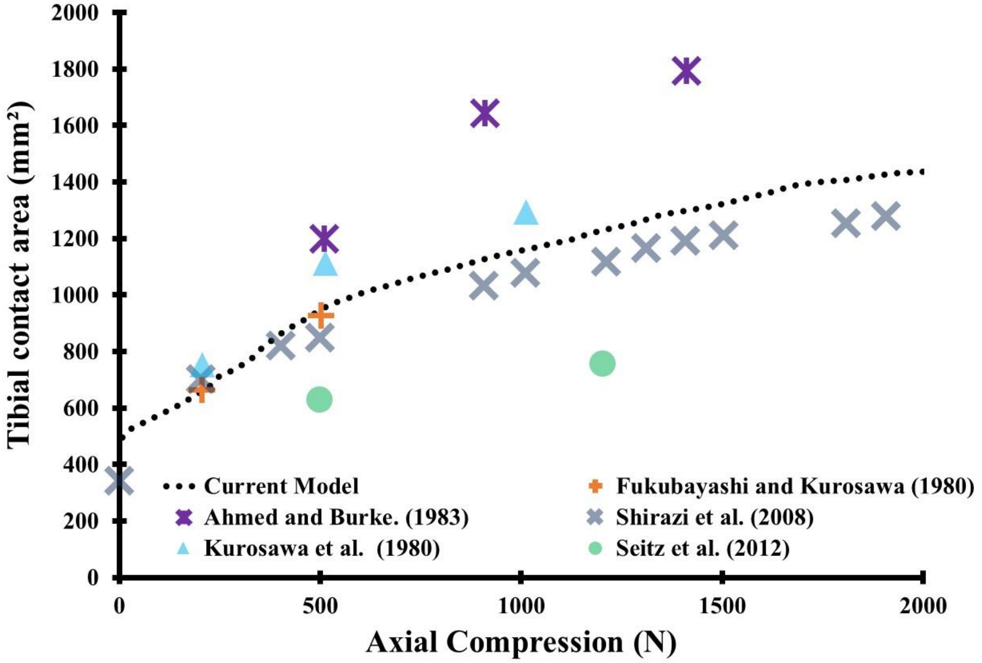

Figure 17: Tibial contact area under axial compressive load up to 2000 N, experimental measurements under almost the same boundary conditions shown for comparison.

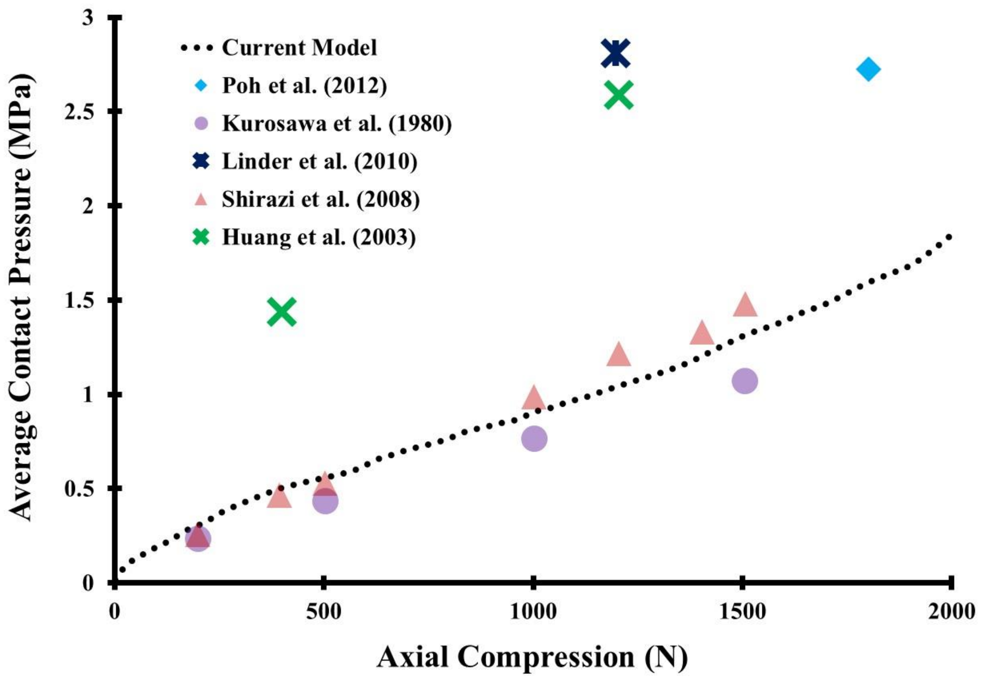

Figure 18: Tibial average contact pressure under axial compressive load up to 2000 N, experimental measurements under almost the same boundary conditions shown for comparison.

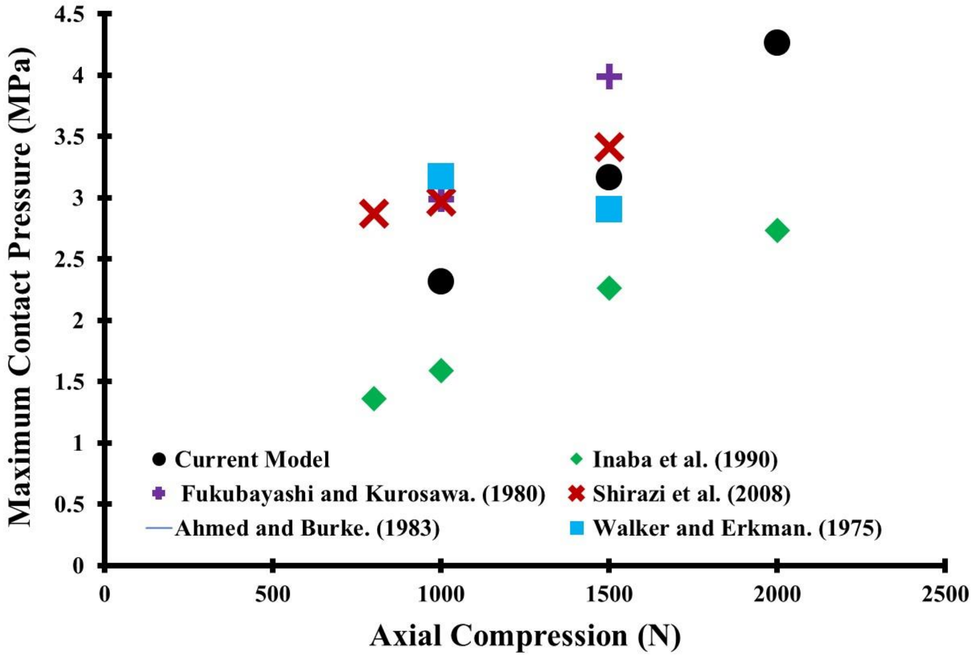

Figure 19: Tibial maximum contact pressure under axial compressive load up to 2000 N, experimental measurements under almost the same boundary conditions shown for comparison.

With intact fibril, our results indicate that cartilage collagen damage was initiated with 32 MPa maximum contact stress and under 5290 N of compressive load (fig. 20). The damage distribution (fibril plastic strain ε_{fp}) of the articular cartilage was restricted to the superficial and middle areas. This damage distribution has been stated in the lateral plateau starting at the same maximum stress concentration area.

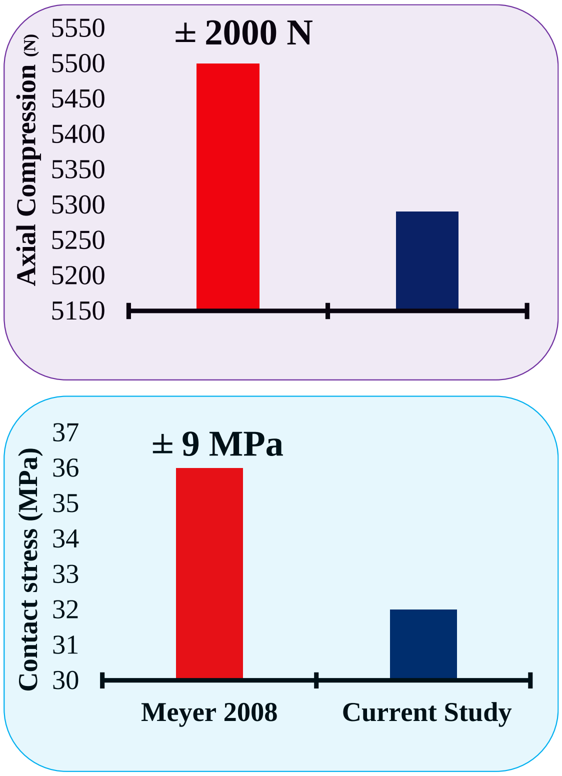

Figure 20: Measured failure axial compression force and contact stress of the articular cartilage[57] along with FE model prediction.

REFERENCES

1 Gautieri, A., Redaelli, A., Buehler, M. J. & Vesentini, S. Age- and diabetes-related nonenzymatic crosslinks in collagen fibrils: candidate amino acids involved in Advanced Glycation End-products. *Matrix Biol* **34**, 89-95, doi:10.1016/j.matbio.2013.09.004 (2014).

2 Orgel, J. P., Irving, T. C., Miller, A. & Wess, T. J. Microfibrillar structure of type I collagen in situ. *Proceedings of the National Academy of Sciences* **103**, 9001-9005 (2006).

3 Zhang, L., Duin, A. C. v., Zybin, S. V. & Goddard Iii, W. A. Thermal decomposition of hydrazines from reactive dynamics using the ReaxFF reactive force field. *The Journal of Physical Chemistry B* **113**, 10770-10778 (2009).

4 Weismiller, M. R., Van Duin, A. C., Lee, J. & Yetter, R. A. ReaxFF reactive force field development and applications for molecular dynamics simulations of ammonia borane dehydrogenation and combustion. *The Journal of Physical Chemistry A* **114**, 5485-5492 (2010).

5 Vanommeslaeghe, K. *et al.* CHARMM general force field: A force field for drug-like molecules compatible with the CHARMM all-atom additive biological force fields. *Journal of computational chemistry* **31**, 671-690 (2010).

6 Chenoweth, K., Van Duin, A. C. & Goddard, W. A. ReaxFF reactive force field for molecular dynamics simulations of hydrocarbon oxidation. *The Journal of Physical Chemistry A* **112**, 1040-1053 (2008).

7 Ebrahimi, S., Ghafoori-Tabrizi, K. & Rafii-Tabar, H. Molecular dynamics simulation of the adhesive behavior of collagen on smooth and randomly rough TiO2 and Al2O3 surfaces. *Computational materials science* **71**, 172-178 (2013).

8 Singam, E. R. A. *et al.* Molecular dynamic simulation studies on the effect of one residue chain staggering on the structure and stability of heterotrimeric collagen-like peptides with interruption. *Biopolymers* **97**, 847-863 (2012).

9 Azhagiya Singam, E., Rajapandian, V. & Subramanian, V. Molecular dynamics simulation study on the interaction of collagen-like peptides with gelatinase-A (MMP-2). *Biopolymers* **101**, 779-794 (2014).

10 Buehler, M. J. Nature designs tough collagen: explaining the nanostructure of collagen fibrils. *Proc Natl Acad Sci U S A* **103**, 12285-12290, doi:10.1073/pnas.0603216103 (2006).

11 Buehler, M. J. Nanomechanics of collagen fibrils under varying cross-link densities: atomistic and continuum studies. *J Mech Behav Biomed Mater* **1**, 59-67, doi:10.1016/j.jmbbm.2007.04.001 (2008).

12 Depalle, B., Qin, Z., Shefelbine, S. J. & Buehler, M. J. Influence of cross-link structure, density and mechanical properties in the mesoscale deformation mechanisms of collagen fibrils. *J Mech Behav Biomed Mater* **52**, 1-13, doi:10.1016/j.jmbbm.2014.07.008 (2015).

13 Depalle, B., Qin, Z., Shefelbine, S. J. & Buehler, M. J. Large Deformation Mechanisms, Plasticity, and Failure of an Individual Collagen Fibril With Different Mineral Content. *J Bone Miner Res* **31**, 380-390, doi:10.1002/jbmr.2705 (2016).

14 Saito, M., Marumo, K., Fujii, K. & Ishioka, N. Single-column high-performance liquid chromatographic–fluorescence detection of immature, mature, and senescent cross-links of collagen. *Analytical biochemistry* **253**, 26-32 (1997).

15 Plimpton, S. Fast parallel algorithms for short-range molecular dynamics. *Journal of computational physics* **117**, 1-19 (1995).

16 Stukowski, A. Visualization and analysis of atomistic simulation data with OVITO–the Open Visualization Tool. *Modelling and Simulation in Materials Science and Engineering* **18**, 015012 (2009).

17 Asaro, R. J. & Rice, J. Strain localization in ductile single crystals. *Journal of the Mechanics and Physics of Solids* **25**, 309-338 (1977).

18 Lee, E. H. Elastic-plastic deformation at finite strains. *Journal of Applied Mechanics* **36**, 1-6 (1969).

19 Buehler, M. J. Nature designs tough collagen: explaining the nanostructure of collagen fibrils. *Proceedings of the National Academy of Sciences* **103**, 12285-12290 (2006).

20 Buehler, M. J. Nanomechanics of collagen fibrils under varying cross-link densities: atomistic and continuum studies. *Journal of the mechanical behavior of biomedical materials* **1**, 59-67 (2008).

21 Tang, H., Buehler, M. J. & Moran, B. A constitutive model of soft tissue: from nanoscale collagen to tissue continuum. *Annals of biomedical engineering* **37**, 1117-1130 (2009).

22 Tang, Y., Ballarini, R., Buehler, M. J. & Eppell, S. J. Deformation micromechanisms of collagen fibrils under uniaxial tension. *Journal of The Royal Society Interface* **7**, 839-850 (2010).

23 Gasser, T. C. & Holzapfel, G. A. A rate-independent elastoplastic constitutive model for biological fiber-reinforced composites at finite strains: continuum basis, algorithmic formulation and finite element implementation. *Computational Mechanics* **29**, 340-360, doi:10.1007/s00466-002-0347-6 (2002).

24 Tang, H., Buehler, M. J. & Moran, B. A constitutive model of soft tissue: from nanoscale collagen to tissue continuum. *Ann Biomed Eng* **37**, 1117-1130, doi:10.1007/s10439-009-9679-0 (2009).

25 Belytschko, T., Liu, W. K., Moran, B. & Elkhodary, K. *Nonlinear Finite Elements for Continua and Structures*. (Wiley, 2014).

26 Guo, Z. Y., Peng, X. Q. & Moran, B. A composites-based hyperelastic constitutive model for soft tissue with application to the human annulus fibrosus. *Journal of the Mechanics and Physics of Solids* **54**, 1952-1971, doi:10.1016/j.jmps.2006.02.006 (2006).

27 Guo, Z. Y., Caner, F., Peng, X. Q. & Moran, B. On constitutive modelling of porous neo-Hookean composites. *Journal of the Mechanics and Physics of Solids* **56**, 2338-2357, doi:10.1016/j.jmps.2007.12.007 (2008).

28 DeBotton, G., Hariton, I. & Socolsky, E. Neo-Hookean fiber-reinforced composites in finite elasticity. *Journal of the Mechanics and Physics of Solids* **54**, 533-559 (2006).

29 Buehler, M. J. & Ballarini, R. *Materiomics: multiscale mechanics of biological materials and structures*. (Springer, 2013).

30 Adouni, M., Mbarki, R., Al Khatib, F. & Eilaghi, A. Multiscale modeling of knee ligament biomechanics. *International Journal for Numerical Methods in Biomedical Engineering* **37**, e3413, doi:https://doi.org/10.1002/cnm.3413 (2021).

31 Sajjadinia, S. S., Haghpanahi, M. & Razi, M. Computational simulation of the multiphasic degeneration of the bone-cartilage unit during osteoarthritis via indentation and unconfined compression tests. *Proceedings of the Institution of Mechanical Engineers, Part H: Journal of Engineering in Medicine* **233**, 871-882, doi:10.1177/0954411919854011 (2019).

32 Natali, A., Pavan, P., Carniel, E. & Dorow, C. A transversally isotropic elasto-damage constitutive model for the periodontal ligament. *Computer Methods in Biomechanics and Biomedical Engineering* **6**, 329-336 (2003).

33 Lemaitre, J. & Desmorat, R. *Engineering damage mechanics: ductile, creep, fatigue and brittle failures*. (Springer Science & Business Media, 2005).

34 Skrzypek, J. J. & Ganczarski, A. *Modeling of material damage and failure of structures: theory and applications*. (Springer Science & Business Media, 2013).

35 Krajcinovic, D. *Damage mechanics*. Vol. 41 (Elsevier, 1996).

36 Volokh, K. Y. Hyperelasticity with softening for modeling materials failure. *Journal of the Mechanics and Physics of Solids* **55**, 2237-2264, doi:https://doi.org/10.1016/j.jmps.2007.02.012 (2007).

37 Volokh, K. Y. Softening hyperelasticity for modeling material failure: Analysis of cavitation in hydrostatic tension. *International Journal of Solids and Structures* **44**, 5043-5055, doi:https://doi.org/10.1016/j.ijsolstr.2006.12.022 (2007).

38 Volokh, K. Y. Nonlinear Elasticity for Modeling Fracture of Isotropic Brittle Solids. *Journal of Applied Mechanics* **71**, 141-143, doi:10.1115/1.1636795 (2004).

39 Bazant, Z. P. & Planas, J. *Fracture and size effect in concrete and other quasibrittle materials*. Vol. 16 (CRC press, 1997).

40 Broberg, K. B. *Cracks and Fracture*. (Elsevier Science, 1999).

41 Hertzberg, R. W., Vinci, R. P. & Hertzberg, J. L. *Deformation and Fracture Mechanics of Engineering Materials, 5th Edition*. (Wiley, 2012).

42 Volokh, K. Y. On modeling failure of rubber-like materials. *Mechanics Research Communications* **37**, 684-689, doi:https://doi.org/10.1016/j.mechrescom.2010.10.006 (2010).

43 Wilson, W., Huyghe, J. M. & van Donkelaar, C. C. Depth-dependent compressive equilibrium properties of articular cartilage explained by its composition. *Biomech Model Mechanobiol* **6**, 43-53, doi:10.1007/s10237-006-0044-z (2007).

44 Fratzl, P. *Collagen: structure and mechanics*. (Springer Science & Business Media, 2008).

45 Kempson, G., Freeman, M. & Swanson, S. Tensile properties of articular cartilage. (1968).

46 Proctor, C., Schmidt, M., Whipple, R., Kelly, M. & Mow, V. Material properties of the normal medial bovine meniscus. *Journal of Orthopaedic Research* **7**, 771-782 (1989).

47 Shirazi, R. & Shirazi-Adl, A. Analysis of articular cartilage as a composite using nonlinear membrane elements for collagen fibrils. *Medical engineering & physics* **27**, 827-835 (2005).

48 Chang, S.-W., Shefelbine, S. J. & Buehler, M. J. Structural and mechanical differences between collagen homo-and heterotrimers: relevance for the molecular origin of brittle bone disease. *Biophysical journal* **102**, 640-648 (2012).

49 Erdemir, A. Open knee: open source modeling & simulation to enable scientific discovery and clinical care in knee biomechanics. *The journal of knee surgery* **29**, 107 (2016).

50 Dhaher, Y. Y. & Kahn, L. E. The effect of vastus medialis forces on patello-femoral contact: a model-based study. *J. Biomech. Eng.* **124**, 758-767 (2002).

51 Donahue, T. L., Hull, M. L., Rashid, M. M. & Jacobs, C. R. A finite element model of the human knee joint for the study of tibio-femoral contact. *J Biomech Eng* **124**, 273-280 (2002).

52 Ahmed, A. & Burke, D. In-Vitro of Measurement of Static Pressure Distribution in Synovial Joints—Part I: Tibial Surface of the Knee. *Journal of Biomechanical Engineering* **105**, 216 (1983).

53 Brown, T. D. & Shaw, D. T. In vitro contact stress distribution on the femoral condyles. *J Orthop Res* **2**, 190-199, doi:10.1002/jor.1100020210 (1984).

54 Poh, S. Y. *et al.* Role of the anterior intermeniscal ligament in tibiofemoral contact mechanics during axial joint loading. *Knee* **19**, 135-139, doi:10.1016/j.knee.2010.12.008 (2012).

55 Seitz, A., Kasisari, R., Claes, L., Ignatius, A. & Durselen, L. Forces acting on the anterior meniscotibial ligaments. *Knee surgery, sports traumatology, arthroscopy : official journal of the ESSKA* **20**, 1488-1495, doi:10.1007/s00167-011-1708-5 (2012).

56 Meyer, E. G., Baumer, T. G., Slade, J. M., Smith, W. E. & Haut, R. C. Tibiofemoral contact pressures and osteochondral microtrauma during anterior cruciate ligament rupture due to excessive compressive loading and internal torque of the human knee. *American Journal of Sports Medicine* **36**, 1966-1977, doi:10.1177/0363546508318046 (2008).

57 Meyer, E. G. & Haut, R. C. Excessive compression of the human tibio-femoral joint causes ACL rupture. *J Biomech* **38**, 2311-2316, doi:10.1016/j.jbiomech.2004.10.003 (2005).
